# CRISPRa‐based activation of *Fgf21* and *Fndc5* ameliorates obesity by promoting adipocytes browning

**DOI:** 10.1002/ctm2.1326

**Published:** 2023-07-18

**Authors:** Hongtao Zhu, Dan Liu, Ming Sui, Meiling Zhou, Beibei Wang, Qinqin Qi, Ting Wang, Guo Zhang, Feng Wan, Bin Zhang

**Affiliations:** ^1^ Department of Neurosurgery Tongji Hospital of Tongji Medical College Huazhong University of Science and Technology Wuhan China; ^2^ Department of Physiology School of Basic Medicine Tongji Medical College Huazhong University of Science and Technology Wuhan China; ^3^ The Institute for Brain Research Collaborative Innovation Center for Brain Science Huazhong University of Science and Technology Wuhan China; ^4^ Key Laboratory of Environmental Health Ministry of Education Department of Toxicology School of Public Health Tongji Medical College Huazhong University of Science and Technology Wuhan China; ^5^ Department of Neurosurgery Guangdong Provincial People’s Hospital, Guangdong Academy of Medical Sciences, Southern Medical University Guangzhou China; ^6^ Hubei Key Laboratory of Drug Target Research and Pharmacodynamic Evaluation Huazhong University of Science and Technology Wuhan China

**Keywords:** adipocyte, browning, CRISPRa, myokines

## Abstract

**Background:**

Skeletal muscle‐secreted myokines widely participate in lipids metabolism through autocrine, paracrine and endocrine actions. The myokines represented by FGF21 and Irisin can promote the browning of adipocytes and serve as promising targets for treating obesity. Although recombinant myokines replacement therapy and AAV (adeno‐associated virus)‐based myokines overexpression have shown a definite effect in ameliorating obesity, novel myokine activation strategies with higher efficacy and safety are still in pressing need. This study aimed to evaluate the therapeutic potential of a novel CRISPR‐based myokines activation strategy in obesity treatments.

**Methods:**

In this study, we used lentivirus and a single AAV vector containing dCas9‐VP64 with a single‐guide RNA to selectively activate *Fgf21* and *Fndc5* expression in skeletal muscles both in vitro and in vivo. The activation efficacy of the CRISPRa system was determined by qRT‐PCR, Western blotting and ELISA. The treatment effect of CRISPR‐based myokines activation was tested in 3T3‐L1‐derived adipocytes and diet‐induced obese (DIO) mice (male C57BL/6 mice, induced at 6‐week‐old for 10 weeks).

**Results:**

The virus upregulates myokines expression in both mRNA and protein levels of muscle cells in vitro and in vivo. Myokines secreted by muscle cells promoted browning of 3T3‐L1‐derived adipocytes. In vivo activation of myokines by AAVs can reduce body weight and fat mass, increase the adipocytes browning and improve glucose tolerance and insulin sensitivity in DIO mice.

**Conclusions:**

Our study provides a novel CRISPR‐based myokines activation strategy that can ameliorate obesity by promoting adipocytes browning.

## Graphical abstract

CRISPRa system induced FGF21 and Irisin expression and secretion in muscle cells.

CRISPRa‐based myokines activation of muscle cells promotes adipocytes browning in vitro.

In vivo CRISPRa‐based activation of myokines ameliorates obesity by promoting adipocytes browning in diet‐induced obese mice.

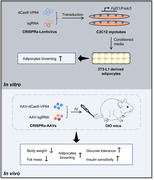

## 1 BACKGROUND

Obesity increases the risk of many metabolic disorders, including type 2 diabetes (T2D) and heart disease, becoming the most common public health issue globally.^1^ As obesity is the increase in the mass of adipose tissue, research interest in adipose tissue is constantly growing. There are two main types of adipose tissues: brown adipose tissue (BAT), which can both store nutrients as lipids and dissipate their energy as heat through non‐shivering thermogenesis, and white adipose tissue (WAT), which stores vast amounts of nutrients as lipids in unilocular white adipocytes.^2^ The beige adipocyte is another well‐explored cell type, which may be derived from WAT precursor cells or transformed from mature WAT. The transformation of beige adipocytes, also known as the ‘browning’ of WAT, correlates with increased energy expenditure.^2^ Thus, the accumulation of beige adipocytes, or browning, becomes a prospective strategy for obesity treatments.^2^

Myokines are signal molecules synthesised and secreted by skeletal muscle.^3^ Myokines are known to regulate numerous processes, including mitochondrial homeostasis, cardiac diseases, cognitive function and bone metabolism; metabolic disorders are no exception.^3^ Among all reported myokines closely associated with adipocytes browning, FGF21 and Irisin are two representatives.^4^ Fibroblast growth factor 21 (FGF21) is secreted by several organs and can act on multiple tissues to regulate energy homeostasis.^5^ Irisin is encoded by fibronectin type III domain‐containing protein 5 (*Fndc5*) gene and secreted by skeletal muscles after cleavage.^6^ Studies have shown that obesity is associated with a decrease in circulating and adipose tissue expression levels of FGF21 and Irisin as well as their specific receptors, β‐Klotho and FGFR1 for FGF21 and ITGA5 for Irisin, respectively.^7^, ^8^, ^9^, ^10^, ^11^ These two cold‐induced myokines drive brown‐fat‐like thermogenesis in murine white fat and serve as potential targets for obesity treatment.^12^ Over the past few years, researchers have tried various treatments based on these two myokines, including recombinant protein injection and in vivo AAV‐mediated gene overexpression.^5^, ^13^, ^14^ However, the existing approaches do not achieve satisfactory effects. The replacement therapy of recombinant protein was subjected to its high cost and poor pharmacokinetic properties. The AAV‐based gene therapy was hampered by excessive upregulation fold and potential abnormal endogenous gene expression induced by random insertions of exogenous gene fragments into the genome.^15^, ^16^, ^17^ New myokine‐based treatments are desperately needed to overcome these obstacles.

Clustered regularly interspaced short palindromic repeats (CRISPR) and RNA‐guided Cas9 nuclease system was first discovered in bacteria and rapidly used in multiplex genome engineering. Beyond editing, dead Cas9 (dCas9) nuclease combined with transcriptional activation or repression effectors can accurately regulate gene expression at the transcriptional level.^18^ The CRISPR–dCas9 system also enables the manipulation of multiple gene targets simultaneously and has been utilised to treat various diseases.^19^, ^20^ Two recent studies investigated the potential preventing effects for obesity in mice by CRISPR‐engineered human adipocytes through gene knockout or transcriptional activation.^21^, ^22^ However, these studies focused on adipose tissues per se. The applications of the CRISPR–dCas9 system in cells other than adipocytes to combat obesity, such as the skeletal muscle‐secreted‐myokine‐based approach, have not been reported yet.

In this study, we utilised the lentivirus and AAV vector‐mediated CRISPR–dCas9‐VP64 (CRISPRa) system to activate myokines gene expression transcriptionally. We confirmed that the CRISPRa system could effectively upregulate the expression and secretion of myokines by muscle cells in vitro and in vivo. Myokines secreted by muscle cells promote the browning of 3T3‐L1‐derived adipocytes. In vivo activation of myokines by AAVs decreased body weight and fat mass, promoted adipocytes browning and ameliorated glucose tolerance and insulin sensitivity in diet‐induced obese (DIO) mice. Our study provides a new perspective on the obesity therapy strategy based on myokines‐mediated muscles‐adipocytes crosstalk.

## 2 METHODS

### 2.1 Animals

Wild‐type C57/BL6J mice were from the Experimental Animal Center of Tongji Medical College, Huazhong University of Science and Technology. The animal room was lit entirely with artificial fluorescent lighting, with a 12 h light–dark cycle (7 AM to 7 PM). Autoclaved tap water and irradiation sterilised chow were provided ad libitum. The obese male mice with C57BL/6 background induced by a high‐fat diet (HFD) and corresponding littermate control mice were purchased from SHULAIBAO Bioscience Inc at the age indicated in the study. The obese mice were fed a 60% fat kcal% HFD (MD12033; Medicience), and the mice in the control group were given 10% fat kcal% normal diet (MD12031; Medicience) for the entire study. All experimental procedures were performed following the Guide for the Care and Use of Laboratory Animals and approved by the University Animal Welfare Committee, Tongji Medical College, Huazhong University of Science and Technology.

### 2.2 Cell cultures

Human embryonic kidney cells (HEK293T), mouse embryonic fibroblasts (NIH‐3T3) and mouse preadipocytes (3T3‐L1) were purchased from China Center for Type Culture Collection and maintained at 37°C and 5% CO_2_ in Dulbecco's modified Eagle's medium (DMEM) containing antibiotics (penicillin and streptomycin sulfate), l‐glutamine, sodium pyruvate and 10% foetal bovine serum (FBS). Mouse C2C12 myoblasts were purchased from ATCC and maintained at 37°C and 5% CO_2_ in DMEM containing antibiotics (penicillin and streptomycin sulphate), l‐glutamine, sodium pyruvate and 20% FBS.

### 2.3 Plasmids

The origins and sequences of plasmids used in this study are shown in Table S1. Primers used for vector construction in this study are shown in Table S2.

### 2.4 Lentivirus package

Lentiviral particles used in this study were produced by transient transfection of plasmids into HEK293T cells and concentrated by PEG8000 (Sigma). HEK293T cells were transfected using PEI max solution (Sigma). In brief, 6 μg of the psPAX2 plasmid, 6 μg of the pMD2.G plasmid and 9 μg of the interested plasmid were transfected using 10× PEI max (1:3 ratio to DNA) into HEK293T cells plated in 10 cm culture dishes. Lentiviral supernatant was collected 72 h after transfection and filtered through a 0.45‐μm filter. The virus was then mixed with 5× PEG8000 at 4°C rotator overnight and the mixture was centrifuged at 5000×*g* for 30 min at 4°C. The lentiviral particles were resuspended with PBS. Cells were infected with concentrated lentivirus containing 10 mg/mL polybrene (Sigma) and then screened using puromycin (Sigma) or G418 (Sigma).

### 2.5 C2C12 myoblast differentiation

As previously described,^23^ C2C12 myoblasts were set up for differentiation on day 0 in 60 mm cell culture dishes at 80% confluence. On day 2, differentiation was induced by replacing the culture medium containing 20% FBS with the differentiation medium containing 2% horse serum, and the media were changed every day afterward. On day 5, 1 nM recombinant agrin was added to stimulate the aggregation of acetylcholine receptors. Sixteen hours after stimulation, cells were washed once with warm PBS and ready for follow‐up experiments.

### 2.6 3T3‐L1 preadipocytes differentiation

Mouse preadipocyte (3T3‐L1) cells were differentiated into mature adipocytes, as described previously.^24^ In brief, preadipocytes were cultured using basal medium (DMEM supplemented with 10% FBS). After 100% confluence (day 2), the cells were initiated for differentiation by change with induction medium 1 (basal medium supplemented with 0.5 mmol/L 3‐isobutyl‐1‐methylxanthine (MedChemExpress; #I5879), 0.25 μmol/L dexamethasone (MedChemExpress; #D4092) and 10 μmol/L insulin (MedChemExpress; HY‐P0035)). Two days later (day 4), the induction medium 1 was changed to induction medium 2 (basal medium supplemented with 10 μmol/L insulin) or conditioned medium of C2C12 with *Fgf21* or *Fndc5* activation for 4 more days. On day 8, mature 3T3‐L1 adipocytes of each group were processed for Oil Red O staining (Sigma; #O0625), qRT‐PCR, immunohistochemical staining and Western blotting to validate differentiation efficiency.

### 2.7 Oxygen consumption rate assay

The oxygen consumption rate (OCR) assay of 3T3‐L1‐derived adipocytes was performed using Oxygen Consumption Assay Kit (Bestbio; #BB‐48211) according to the manufacturer's instructions. Briefly, 3T3‐L1 preadipocytes were seeded in black 96‐well cell culture plates with clear bottom and differentiation according to previous protocols. After treatment with conditioned medium of C2C12 cells with myokines activation, the BBoxiProbeTM R01 oxygen fluorescence probes were added into wells and then oxygen sealing solution was used to isolate oxygen. The fluorescence intensity was measured by a PerkinElmer EnSpire multimode plate reader over 90 min at 2 min intervals.

### 2.8 Primary adipocytes isolation and differentiation

Primary mice adipocytes isolation and differentiation were done as described previously.^25^ Briefly, the subcutaneous adipose tissues of male C57/BL6J mice (4 weeks old) were collected and finely minced with scissors. Then, the tissues were resuspended and digested in 2 mg/mL Collagenase I (Solarbio; #C8140) at 37°C for 20 min. The digestion was terminated by mixing with an equal volume of basal medium (DMEM supplemented with 10% FBS) and centrifuged at 2000 rpm for 10 min. The pellet was re‐suspended in basal medium and passed through a 40 μm cell strainer. The cell suspension was re‐centrifuged and re‐suspended in basal medium and incubated in 5% CO_2_ at 37°C. After 6 h incubation, the cells were washed three times with PBS to remove floating cells. The differentiation of primary adipocytes was the same with 3T3‐L1 preadipocytes as mentioned ahead.

### 2.9 AAV production and delivery

The AAV was produced using triple transfection of plasmids to HEK293T cells and purified by AAV Purification Miniprep Kit (Biomiga: #BW‐V1269‐00). Briefly, HEK293T cells were transfected using 10× PEI max. The helper plasmid, capsid and plasmid of interest were transfected at a 2:1:1 ratio. The supernatants and cells were collected 72 h after transfection. The virus was purified and concentrated following the instruction manual of the AAV Purification Miniprep Kit. Finally, the virus was tittering by SYBR Green qRT‐PCR as reported previously.^26^ AAV‐dCas9‐VP64‐EGFP (10^12^ vg) and AAV‐sgRNAs (10^12^ vg) were 1:1 mixed in a total 100 μL volume and delivered into mice via tail vein injection.

### 2.10 RNA isolation and real‐time PCR

The total RNA of cells and tissues were isolated using RNA isolater Total RNA Extraction Reagent (Vazyme) according to the instruction manual. The concentration and integrality of total RNA were detected by NanoDrop Spectrophotometer (ThermoFisher) and agarose gel electrophoresis. Synthesis of cDNA was performed in a reaction system of 20 μL containing 1 μg total RNA, gDNA wiper Mix and HiScript III qRT SuperMix following the instruction manual of HiScript® II Q Select RT SuperMix for qPCR (Vazyme) and stored at −20°C. Real‐time PCR was performed on StepOnePlus Real‐Time PCR Systems (ThermoFisher) using ChamQ SYBR qPCR Master Mix (Vazyme). The relative mRNA expression level of genes was calculated using the 2^−ΔΔCT^ method. The primers used for real‐time PCR assays in this study are shown in Table S4.

### 2.11 Immunoblotting

Total proteins of cells and tissues were extracted using RIPA lysis buffer containing protease and phosphatase inhibitor cocktail (MedChemExpress). The protein concentrations were determined by the BCA kit (Beyotime), and 40−60 μg of denatured proteins were separated by SDS‐PAGE gels and transferred onto PVDF membranes (Bio‐Rad). The detection of protein signals was performed using ECL reagent (Bio‐Rad). The primary antibodies involving against FGF21 (1:1000; Abcam; Cat# ab171941, RRID: AB_2629460), FNDC5 (1:1000; Proteintech; Cat# 23995‐1‐AP, RRID: AB_2879394), UCP1 (1:1000; ABclonal; Cat# A5857, RRID: AB_2766607), β‐actin (1:5000; ABclonal; Cat# AC038, RRID: AB_2863784), α‐tubulin (1:5000; ABclonal; Cat# AC007, RRID: AB_2772755) and HRP conjugated goat anti‐mouse and goat anti‐rabbit secondary antibody (1:10 000; Jackson ImmunoResearch) were used.

### 2.12 Immunohistochemistry staining

Brown adipose tissues of mice were fixed in fat‐specific fixative (Servicebio) for 48 h and processed for paraffin embedding and section. The slides were deparaffinised, hydrated and boiled in Citrate Antigen Retrieval Solution pH6.0 (ZSGB‐Bio) to perform antigen retrieval. After being treated with 3% peroxidase (H_2_O_2_) for 15 min at room temperature, the slides were incubated with primary antibody overnight at 4°C and secondary antibody for 1 h at room temperature. DAB Substrate kit (ZSGB‐Bio) was used to show positive staining, followed by counterstaining with haematoxylin, dehydration and mounting. The primary antibodies used in the immunohistochemistry staining in this study were UCP1 (1:100; ABclonal; Cat# A5857, RRID: AB_2766607).

### 2.13 Intraperitoneal glucose tolerance and intraperitoneal insulin tolerance tests

Intraperitoneal insulin tolerance test (IPITT) and intraperitoneal glucose tolerance test (IPGTT) were performed to evaluate the glucose tolerance and insulin sensitivity of mice.^27^ To closely mimic the clinically used examinations for human, we performed mice fasting from 7:00 AM to 3:00 PM and injected with 2 g/kg (glucose/body weight) sterile glucose solution (Sigma) for the IPGTT and 1 U/kg (insulin/body weight) insulin solution (Novo Nordisk) for the IPITT. After injection, tail blood glucose levels of mice were monitored using a handheld glucometer (Yuwell) at 0, 15, 30, 60 and 90 min for IPGTT and 0, 15, 30, 60, 90 and 120 min for IPITT. Inject glucose immediately to avoid mortality if the mice appear ill during IPITT.

### 2.14 Body composition analysis

Body composition was analysed by Minispec mq‐one Series TD‐NMR Analyzer (Bruker BioSpin) based on nuclear magnetic resonance technology. The body weights of test animals were measured before the experiments. Animals were put into Minispec probe after analyser calibration. The contents of each animal's fat, lean tissues and free body fluid were measured for subsequent analysis.

### 2.15 Food intake and body weight measurements

The mice were housed in clean cages singly with adequate food and water, and the body weight and food intake of each mouse were measured every 2 days.

### 2.16 Statistical analysis

Statistical analysis was performed using GraphPad Prism 7.0 software. All data were displayed as mean ± standard error of the mean. Unpaired two‐tailed Student's *t*‐test was used for differential analysis of two groups, and two‐way analysis of variance (ANOVA) was used for multiple comparisons. Statistical significance was defined as *p* < .05. All experiments were repeated three or more times.

## 3 RESULTS

### 3.1 CRISPRa system significantly upregulates myokines expression and secretion by muscle cells

To activate the expression of myokines in skeletal muscles using the CRISPRa system, we first clarify the endogenous expression and secretion of FGF21 and Irisin by skeletal muscle cells. C2C12 myocytes were differentiated into myotubes, and recombinant argin protein was applied to induce AChR clustering to test the functional maturation of the skeletal muscle cells (Figure S1A). The morphology and fluorescent field of differentiated C2C12 myotubes stained by α‐BTX showed integrated tubular structure and AChR clustering on myotubes (Figure S1B). The levels of mRNA, protein expression and secretion from D0 to D5 of the C2C12 cells were examined (Figures S1C–J). The results showed that these two myokines were expressed at both the mRNA and protein levels and secreted by skeletal muscle cells endogenously, as previously reported.^28^, ^29^

**FIGURE 1:**
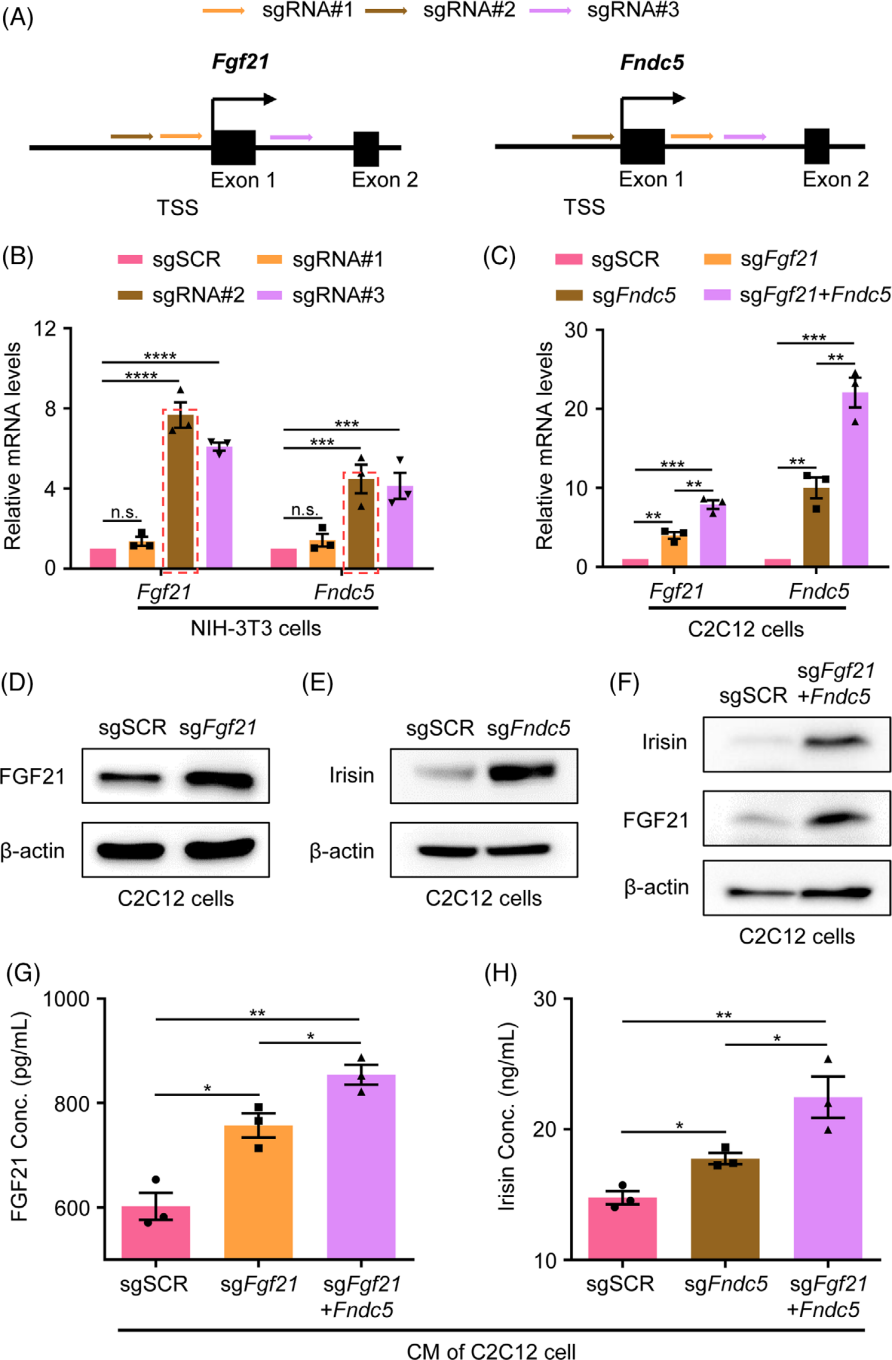
The CRISPRa system significantly upregulates myokines expression and secretion in muscle cells. (A) Diagram of the sgRNA design of *Fgf21* and *Fndc5*. (B) Quantification of *Fgf21* and *Fndc5* activation by qRT‐PCR using dCas9‐VP64 with three different sgRNAs in NIH‐3T3 cells. (C) Quantification of *Fgf21* and *Fndc5* activation by qRT‐PCR using dCas9‐VP64 with selected sg*Fgf21*, sgFndc5 and sg*Fgf21*+*Fndc5* in C2C12 cells. (D–F) Western blot assay shows the upregulated protein level of FGF21 and Irisin using dCas9‐VP64 with single sg*Fgf21*/sg*Fndc5* and sg*Fgf21*+*Fndc5* in C2C12 cell lysates. (G and H) ELISA assay shows upregulation of secretory FGF21 and Irisin using dCas9‐VP64 with single sg*Fgf21*/sg*Fndc5* and sg*Fgf21*+*Fndc5* in C2C12 conditioned media.

Next, we used the CRISPRa system to activate myokine expression in C2C12 cells. Three sgRNAs targeting different sites around the transcription start site (TSS) of *Fgf21* and *Fndc5* were designed, respectively (Figure 1A). The mouse fibroblast cell line, NIH‐3T3, was used to verify the activation efficiency of these sgRNAs. As shown in Figure 1B, the sgRNAs targeting different sites exhibited different activation efficiency, and the sgRNA with the greatest efficiency (sg*Fgf21*#2 and sg*Fndc5*#2, referred to as sg*Fgf21* and sg*Fndc5* hereafter) was selected for further validation and in vitro and in vivo experiments. Since FGF21 and Irisin may have synergistic effects in promoting adipocytes browning,^12^ the construct containing two sgRNAs with the best efficiency targeting *Fgf21* and *Fndc5* simultaneously was also generated to explore the feasibility of regulating the expression of two genes with one vector. We found that these two selected sgRNA could effectively activate *Fgf21* and *Fndc5* mRNA expression in C2C12 cells individually or concurrently, compared with control cells with sgSCR (a non‐targeting scramble sgRNA) (Figure 1C). The protein levels of FGF21 and Irisin in cell lysates were upregulated in C2C12 cells transduced with the CRISPRa system targeting a single myokine gene and two myokine genes concurrently, as shown by western blotting (Figures 1D–F). Moreover, we collected the conditioned media of C2C12 cells of each group, and ELISA tests showed the secretion of FGF21 and Irisin were enhanced in C2C12 cells with myokines activation as well (Figures 1G and H). Interestingly, coactivation of *Fgf21* and *Fndc5* yield higher efficiency in both mRNA and protein levels than single myokine activation. We believe that this phenomenon may root in the synergistic effects of FGF21 and irisin on muscle cell function. Since myokines could regulate skeletal muscle maturation and function,^30^ we also examined whether myocyte differentiation was affected by myokine genes activation. Our results show that activation of myokines in C2C12 cells enhanced muscle‐specific genes, including *Myf5*, *Myd*, *Myg* and *Mck*, expression, suggesting that the CRISPRa‐based myokines activation could promote myocyte differentiation (Figure S2). These results indicate that the CRISPRa system significantly upregulates endogenous myokines expression and secretion with biological function in muscle cells.

### 3.2 Conditioned media of muscle cells with myokines activation inhibits fat accumulation in adipocytes in vitro

**FIGURE 2:**
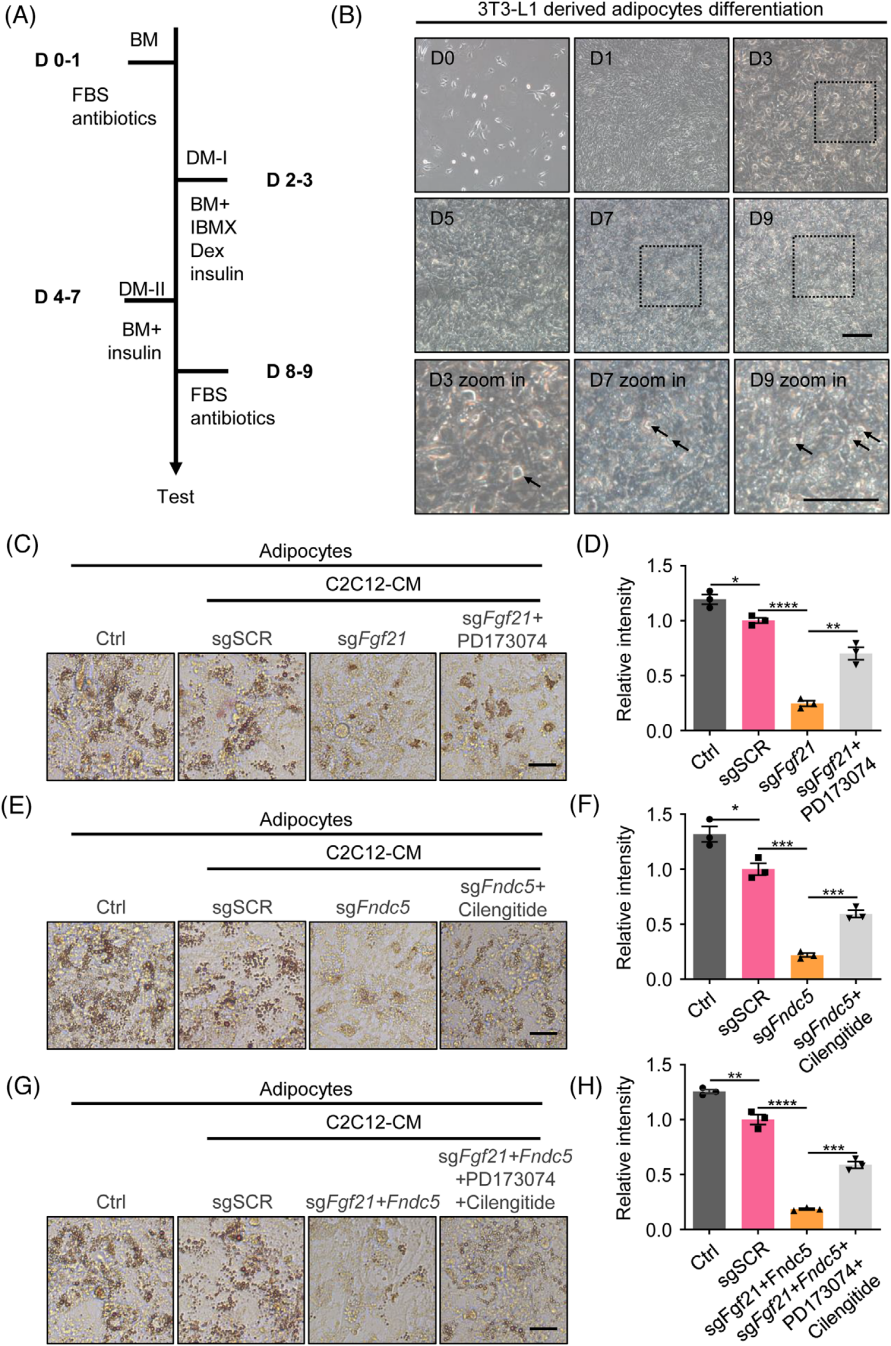
Conditioned media of muscle cells with myokines activation inhibits fat accumulation in adipocytes in vitro. (A) Protocols for 3T3‐L1 adipocyte differentiation. (B) The representative figure for different time points of 3T3‐L1 adipocyte differentiation. Confluent monolayers (D0–D1) were treated with dexamethasone, methylisobutylxanthine (IBMX) and insulin (D2–D3). After 48 h of treatment, cells were treated with fresh or conditioned media with insulin alone for 4 days. Finally, cells were cultured with fresh DMEM complete medium for 2 days. The arrows indicate the fat droplets. (C) Oli Red O staining of 3T3‐L1 derived adipocytes stimulated by conditioned media of C2C12 cells with activation of *Fgf21* (with or without FGFR1 inhibitor PD173074) and the relative intensity of lipid droplets (D) (*n* = 3). (E) Oli Red O staining of 3T3‐L1 derived adipocytes stimulated by conditioned media of C2C12 cells with activation of *Fndc5* (with or without Integrin αVβ5 inhibitor Cilengitide) and the relative intensity of lipid droplets (F) (*n* = 3). (G) Oli Red O staining of 3T3‐L1 derived adipocytes stimulated by conditioned media of C2C12 cells with activation of *Fgf21* and *Fndc5* (with or without FGFR1 inhibitor PD173074 and Integrin αVβ5 inhibitor Cilengitide) and the relative intensity of lipid droplets (H) (*n* = 3). Scale bars in b, 100 μm, c, e and g, 25 μm.

Skeletal muscle is considered an endocrine organ, and myokines are involved in whole‐body physiological functions in autocrine, paracrine and endocrine ways.^30^, ^31^, ^32^ Myokines‐mediated muscle‐adipocyte interactions are believed to play a significant role in the regulation of adipocytes.^33^ To further examine the biological activity of overexpressed myokines, we first collected conditioned media of C2C12 cells with myokines activation after confirming that the CRISPRa system effectively upregulates myokines expression and secretion in these muscle cells. The 3T3‐L1 preadipocytes and primary adipose‐derived stromal vascular fraction cells (SVF cells) were used as in vitro model of adipocyte differentiation (Figure 2A) as previously reported.^24^ After induction with 3‐isobutyl‐1‐methylxanthine, dexamethasone and insulin, extensive fat droplets accumulated at D9 post‐differentiation, demonstrating successful adipocyte differentiation (Figure 2B). Next, we treated the 3T3‐L1‐derived adipocytes and SVF‐derived adipocytes with the conditioned media of C2C12 cells on day 4 post‐induction to investigate the impact of myokines activation on adipocytes. For adipocytes in each group, Oil Red O staining was performed to show fats. Adipocytes treated with conditioned media of C2C12 cells with myokines activation show fewer fats (as revealed by Oil Red O staining) than wild‐type adipocytes and adipocytes in the control group treated with conditioned media of C2C12 with CRISPRa‐sgSCR (Figures 2C–H and S3A–F). Moreover, to confirm that the effect of conditioned media of C2C12 cells with myokines activation on adipocytes is due to the increased myokines, we administered FGFR1 (receptor of FGF21) inhibitor PD173074 and Integrin αVβ5 (receptor of Irisin) inhibitor Cilengitide along with C2C12 conditioned medium with myokines activation. The inhibitors reversed the effect of conditioned media of C2C12 cells with myokines activation on adipocytes, which indicates the effects shown are the result of the crosstalk between skeletal muscle cells‐adipocytes (Figures 2C–H and S3A–F). In conclusion, myokines activation in C2C12 cells mediated by CRISPRa could inhibit fat accumulation in vitro.

### 3.3 Conditioned media of muscle cells with myokines activation promotes adipocytes browning in vitro

**FIGURE 3:**
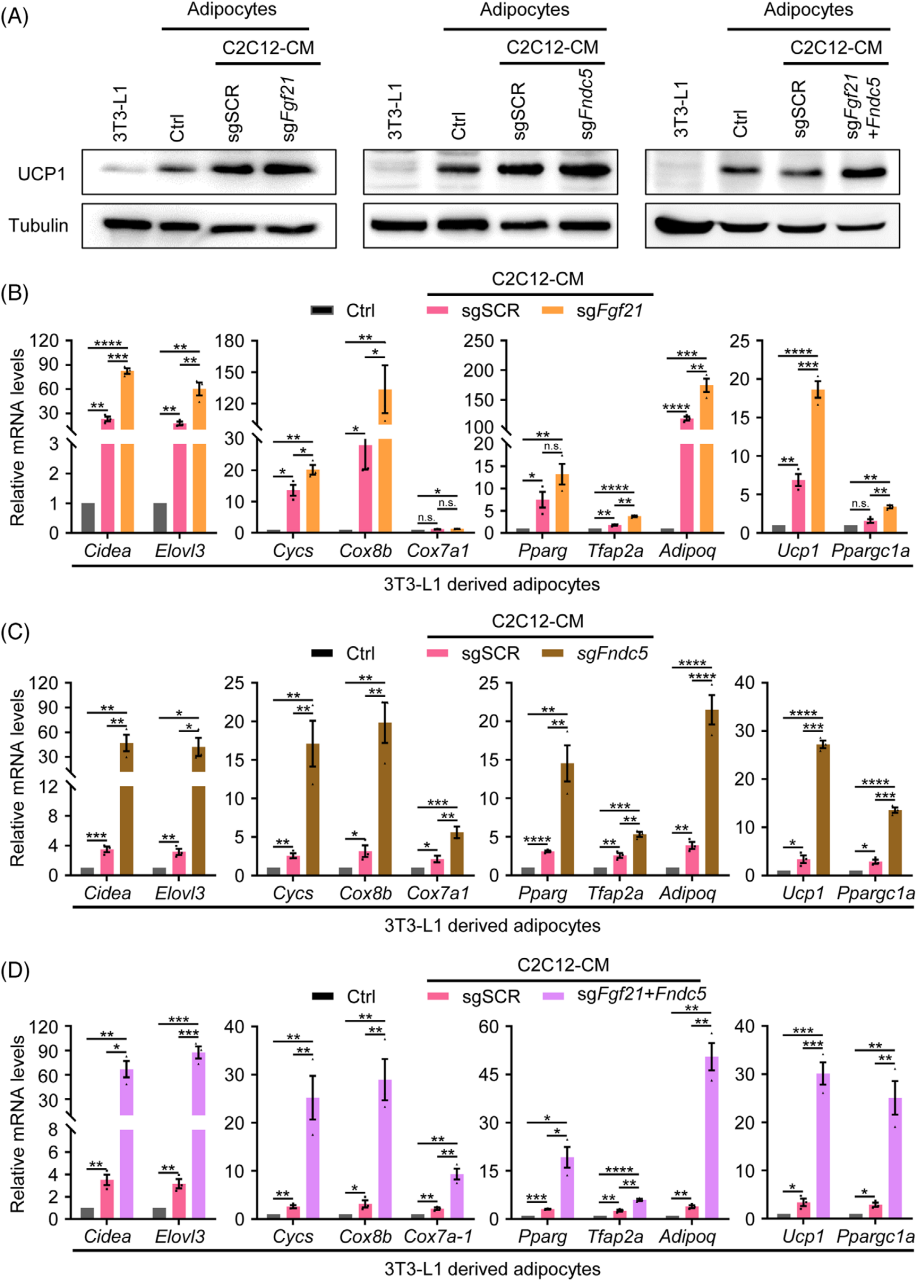
Conditioned media of muscle cells with myokines activation promote adipocytes browning in vitro. (A) Western blot assay shows the level of UCP1 protein in 3T3‐L1 preadipocytes and 3T3‐L1 derived adipocytes with activation of *Fgf21*/*Fndc5*/*Fgf21*+*Fndc5*. Tubulin was used as an internal reference. (B–D) qRT‐PCR quantification of BAT‐selective genes (*Cidea*, *Elovl3*), mitochondrial genes (*cyc*, *cox8b*, *cox7a1*), adipocyte markers (*Pparg*, *Tfap2a*, *Adipoq*) and thermogenesis‐related genes (*Ucp1*, *Ppargc1a*) in 3T3‐L1 preadipocytes and 3T3‐L1‐derived adipocytes treated with conditioned media of *Fgf21*/*Fndc5*/*Fgf21*+*Fndc5*‐activated C2C12 cells.

The therapeutic potential of BAT in endocrine and metabolic disorders has been widely investigated.^34^ BAT is enriched with mitochondria containing the inner mitochondrial proton carrier uncoupling protein 1 (UCP1) that can uncouple oxidative phosphorylation, thus promoting thermogenesis and increasing metabolic rate.^35^ We set to further explore the effects of myokines activation on adipocytes browning. The protein level of UCP1 (Figure 3A), the OCR (Figure S4C) and the mRNA level of BAT‐selective genes (*Cidea*, *Elovl3*), mitochondrial genes (*Cyc*, *Cox8b*, *Cox7a1*), adipocytes markers (*Pparg*, *Tfap2a*, *Adipoq*) and thermogenesis‐related genes (*Ucp1*, *Pgc1α*) in 3T3‐L1 derived adipocytes stimulated by the conditioned media of C2C12 cells with myokines activation were determined by Western blot and qRT‐PCR assays. The results show that conditioned media of muscle cells with activation of single or combined myokines significantly upregulated protein level of UCP1, the oxygen consumption and mRNA level of *Elovl3, Cidea, cyc, cox8b, cox7a1, Pparg*, *Tfap2a*, *Adipoq, Pgc1α* and *Ucp1* genes (Figures 3B–D). Moreover, we also administered FGFR1 (receptor of FGF21) inhibitor PD173074 and Integrin αVβ5 (receptor of Irisin) inhibitor Cilengitide along with C2C12 conditioned medium with myokines activation and detect the protein level of UCP1 and the OCR of adipocytes in each group. The effects of conditioned media of C2C12 cells with myokines activation on UCP1 expression and the OCR of adipocytes were also reversed by the inhibitors (Figures S4A–C). Together, these results indicate that conditioned media of C2C12 cells with myokines activation could promote adipocytes browning in vitro.

### 3.4 CRISPRa system effectively upregulates myokines expression and secretion and decreases body weight and body fat in DIO mice

Adeno‐associated virus (AAV) delivery of the CRISPR system for in vivo genome editing and transcriptionally regulation of gene expression has been applied in treating various diseases, including transthyretin amyloidosis, Duchenne muscular dystrophy, mucopolysaccharidosis, Pompe disease and Huntington's disease.^36^, ^37^, ^38^, ^39^, ^40^ AAV is a large family with multiple serotypes suitable for certain in vivo gene therapy applications.^41^ Among the various serotypes, AAV9 has been used successfully in the treatment of systemic diseases and muscle‐related diseases.^42^, ^43^ We packed dCas9‐VP64 and sgRNA into AAV9 using triple‐plasmid transfection methods, as evidenced by agarose gel electrophoresis (Figure S5). To determine the appropriate titer of the modified AAV9 with effective transduction and minimal side effects, we first administered the AAV9‐dCas9‐VP64 virus with gradient titres to healthy C57BL/6 mice by tail vein injection, and mice were sacrificed for analysis 9 weeks after injection (Figure S6A). The fluorescent images of the mouse organs showed that mice injected with 10^12^ vg virus achieved the expected efficiency of infection (Figures S6B–D). After in vivo verification of AAV9 titres, we injected the CRISPRa system containing AAV9‐dCas9‐VP64 and AAV9‐sgRNA into healthy C57BL/6 mice (Figure S7A), and the fluorescent images of mouse organs showed successful delivery of dCas9‐VP64 and sgRNA simultaneously by AAV9 (Figure S8). qRT‐PCR and ELISA assays suggested that the CRISPRa system effectively upregulated myokines expression (anterior tibial muscle) and secretion (serum) of skeletal muscles in healthy C57BL/6 mice at 4 weeks post‐injection (Figures S7B–E). Furthermore, AAV9‐mediated delivery of the CRISPRa system exhibited well safety, as C57BL/6 mice administered with AAVs had normal physiological functions, and all organs showed normal morphology and size at 9 weeks after injection.

**FIGURE 4:**
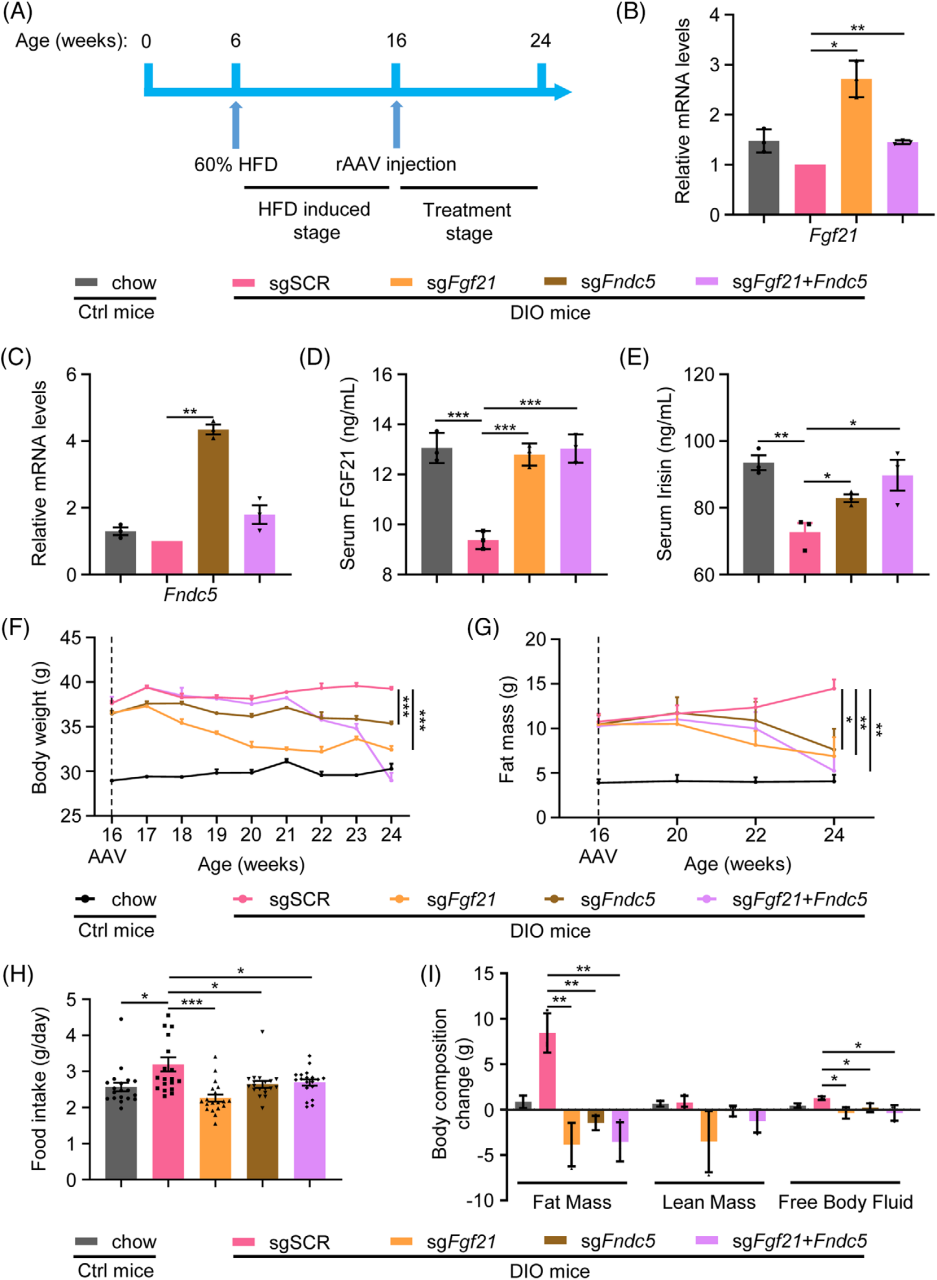
CRISPRa system effectively upregulates myokines expression and secretion, decreases body weight and body fat in DIO mice. (A) Schematic protocol for DIO mice establishment and treatment. Obese mice were fed with 60% fat kcal% forage for 10 weeks and then administered with 10^12^ vg/mouse of AAV9‐CRISPRa vectors. Control chow‐fed mice with 10% fat kcal% forage. (B and C) qRT‐PCR quantification of the mRNA expression of *Fgf21* in the muscle of chow and DIO mice 2 months after vector administration. The mRNA levels were normalised to sgSCR group. (D and E) Circulating levels of FGF21 and Irisin in mouse serum from each group were measured by ELISA 2 months after vector administration. (F) Weekly weight measurement of chow and DIO mice treated with AAV9. The measurement was started at the time of AAV9 vector injection (at 16 weeks of age). (G) Fat mass in each group at 0 (*n* = 9–12 mice per group), 4 (*n* = 9–12 mice per group), 6 (*n* = 9–11 mice per group), 8 (*n* = 3–7 mice per group) weeks post‐injection under a long‐time chow diet and HFD. (H) Histogram depicting the food intake of chow‐fed or HFD‐fed mice and administered with AAV9‐CRISPRa. (I) Changes in body composition of chow mice and DIO mice administered with AAV9‐CRISPRa at 8 weeks post‐injection. The fat mass, lean mass and free body fluid of each mouse were measured and analysed.

Next, we generated DIO mice through HFD feeding to investigate the functions of the CRISPRa system in the obesity model (Figure 4A). When provided with the HFD for 10 weeks (from week 6 to week 16), DIO mice exhibited higher body weight, more food intake, higher fat mass and lean mass, subscapular‐subcutaneous (back) and inguinal‐perigonadal (abdomen) fat deposits compared to chow mice (Figure S9). DIO mice also exhibited low expression and secretion of FGF21, Irisin (Figures S10A–D) along with low UCP1 expression (Figures S10E–G). The AAV9‐CRISPRa viruses were then injected into DIO mice through the tail vein at week 16 (Figure 4A). Using the qRT‐PCR and ELISA assays, we found that the CRISPRa system effectively upregulates myokines expression and secretion in DIO mice (Figures 4B–E). Furthermore, as revealed by regular monitoring, CRISPRa injection significantly reduced the body weight and food intake of DIO mice. In particular, the body weights of mice in the group injected with the sg*Fgf21* virus decreased by approximately 40% compared to mice in the control group (Figures 4F and H). Body composition measurements also showed a profound AAV9‐CRISPRa‐induced total body fat mass reduction in sg*Fgf21*, sg*Fndc5* and sg*Fgf21*+sg*Fndc5* groups compared with the control group (Figures 4G and I).

### 3.5 In vivo CRISPRa‐based activation of myokines promotes adipocytes browning, improves HFD‐associated WAT hypertrophy, hepatic steatosis, inflammation and fibrosis in DIO mice

**FIGURE 5:**
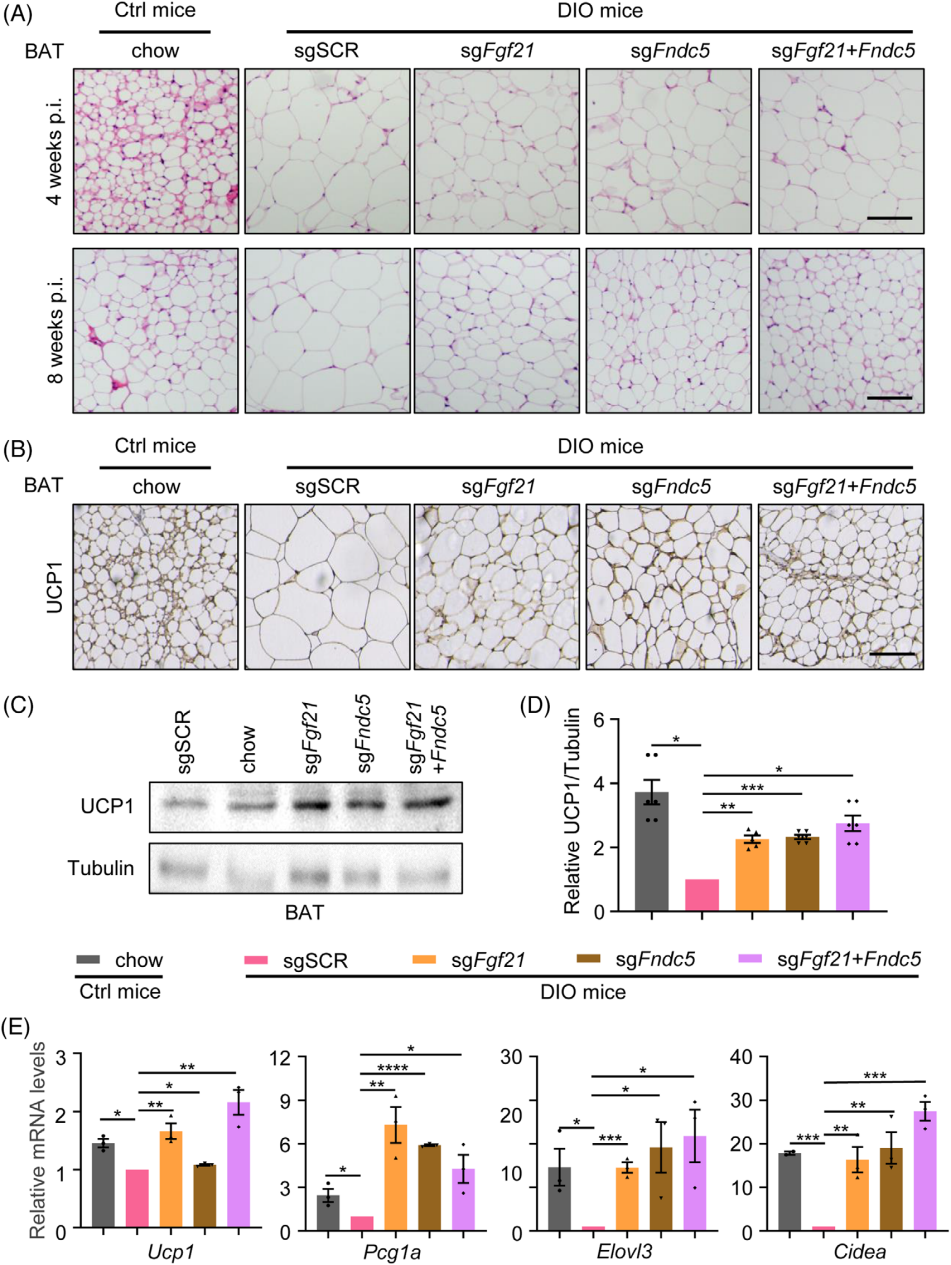
In vivo CRISPRa‐based activation of myokines promotes adipocytes browning in DIO mice. (A) H&E staining shows reduced lipid content in BAT of mice with CRISPRa‐based myokines activation at 4‐ and 8‐weeks post‐injection. Images are representative of four sections of three mice in each group. (B) Immunohistochemical staining for UCP1 (BAT marker) of BAT sections from chow or HFD mice that received AAV9‐CRISPRa vectors. (C) Western blot analysis of the UCP1 protein content in BAT from mice in each group. (D) The histogram depicts the densitometric analysis of immunoblots in C. (E) qRT‐PCR quantification of markers specific to BAT (*Elovl3*, *Cidea*) and thermogenesis (*Ucp1*, *Ppargc1a*) in BAT of mice in each group. The mRNA levels were normalised to sgSCR group. Scale bar in A and B, 100 μm.

Since DIO mice exhibit low myokines expression and secretion and low UCP1 expression (Figure S10), we wondered if CRISPRa system‐mediated myokines activation can rescue those manifestations. Haematoxylin and oeosin (H&E) and immunohistochemical staining of BAT in each group showed decreased content of adipocytes and elevated UCP1 expression in the myokines activation group at 4‐ and 8‐weeks post virus injection (Figures 5A and B). The upregulated protein level of UCP1 and the enhanced mRNA level of BAT and thermogenesis‐specific markers were confirmed by Western blotting and qRT‐PCR (Figures 5C–E). These results demonstrate that CRISPRa‐based myokines activation could decrease body weight and body fat and promote adipocytes browning in DIO mice.

**FIGURE 6:**
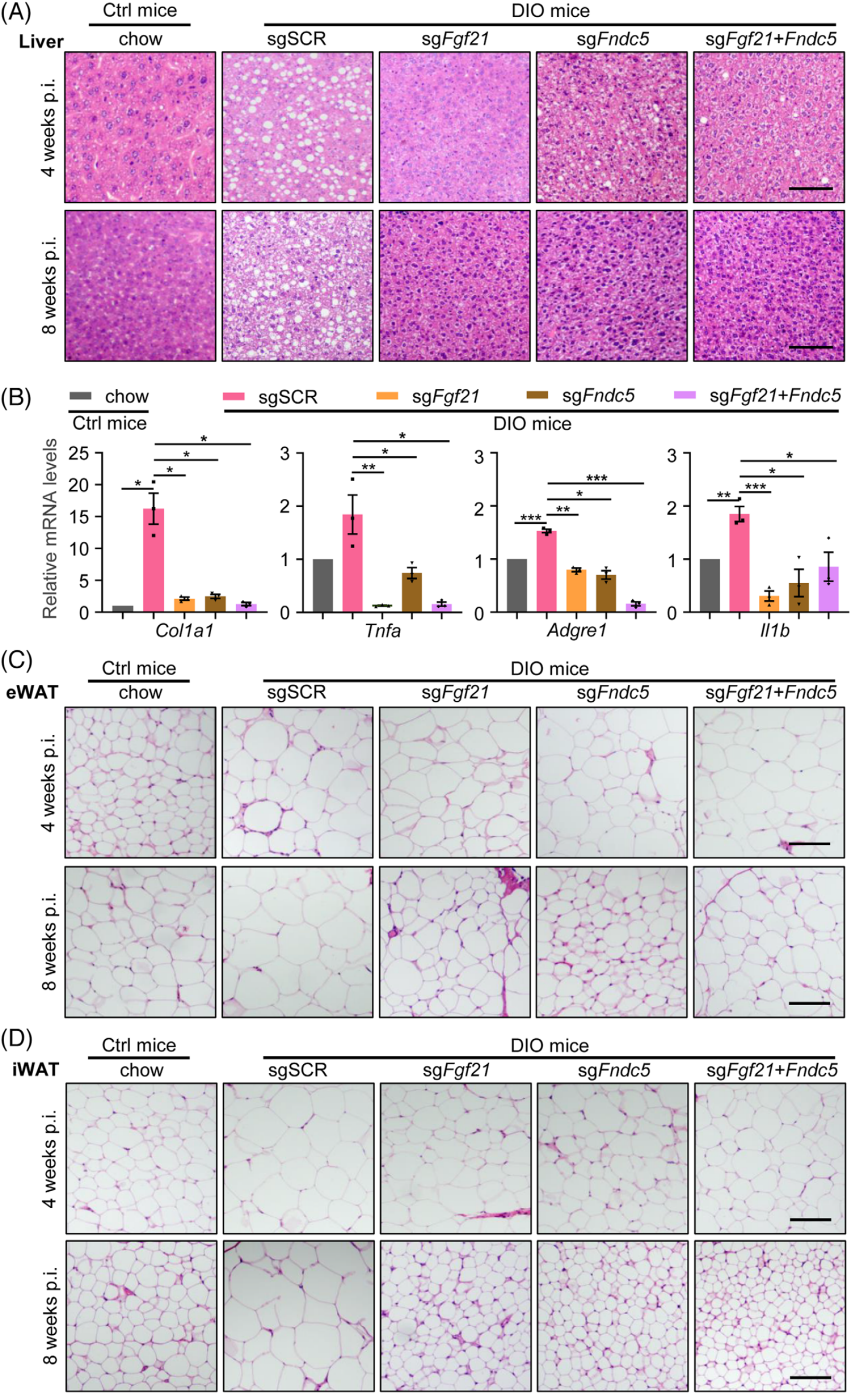
In vivo CRISPRa‐based myokines activation improves HFD‐associated WAT hypertrophy, hepatic steatosis, inflammation and fibrosis in DIO mice. (A) Representative images of H&E staining of liver sections were obtained from animals fed as chow or as HFD and administered with AAV9‐CRISPRa after 4 and 8 weeks. HFD induced the deposition of lipid droplets in the liver, which was reverted by AAV9‐CRISPRa‐based myokine activation. (B) qRT‐PCR quantification of the mRNA expression of *Col1a1* and the markers of inflammation *Tnfa*, *Adgre1* and *Il1b* in the liver tissue from mice in each group. (C and D) Representative images of the H&E staining of the eWAT (C) and iWAT (D) from animals fed as chow or as HFD and administered with 10^12^ vg/mouse AAV9‐CRISPRa vectors at 4 weeks and 8 weeks post‐injection. Scale bar in A, C, D, 100 μm.

Moreover, H&E staining of the liver and WAT showed that DIO mice had marked hepatic steatosis and WAT hypertrophy compared with chow mice (Figure S11A and 9F–I). In contrast, DIO mice treated by AAV9‐CRISPRa showed evidenced reversal of the pathological deposition of lipids in the liver and relieved WAT hypertrophy (Figures 6A, C, D and S12). DIO mice also display hepatic inflammation and fibrosis (Figures S11B–E), and these symptoms were improved by myokines activation, as the expression of key genes involved in hepatic inflammation, including *Col1a1*, *Tnfa*, *Adgre1* and *Il1b*, decreased significantly in the liver of animals (Figure 6B).

### 3.6 In vivo CRISPRa‐based activation of myokines ameliorates glucose tolerance and insulin sensitivity in DIO mice

**FIGURE 7:**
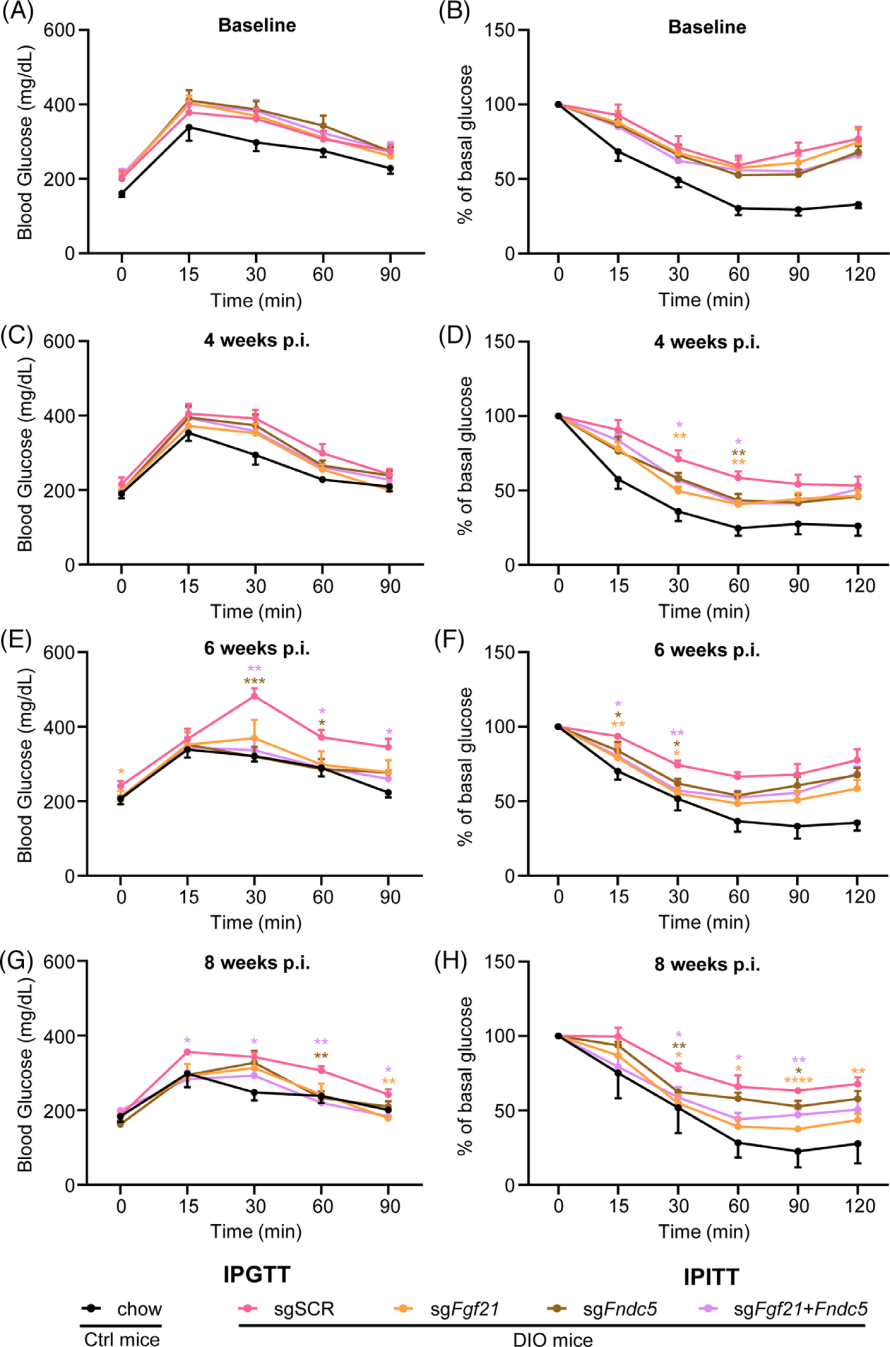
In vivo CRISPRa‐based myokines activation ameliorates glucose tolerance and insulin sensitivity in DIO mice. Glucose tolerance (left) was studied in all experimental groups after an intraperitoneal injection of glucose (2 g/kg body weight). Insulin sensitivity was determined in the group of mice after an intraperitoneal injection of insulin (1.2 units/kg body weight). Results were calculated as the percentage of initial blood glucose levels. Tests were performed at baseline (A and B) and 4 weeks (C and D), 6 weeks (E and F) and 8 weeks (G and H) post AAV injection on chow and DIO mice. All data represent the mean ± SEM. In (A), chow (*n* = 12), AAV9‐CRISPRa‐sgSCR (*n* = 12), AAV9‐CRISPRa‐sg*Fgf21* (*n* = 12), AAV9‐CRISPRa‐sg*Fndc5* (*n* = 12), AAV9‐CRISPRa‐sg*Fgf21*+*Fndc5* (*n* = 11). In (B), chow (*n* = 12), AAV9‐CRISPRa‐sgSCR (*n* = 11), AAV9‐CRISPRa‐sg*Fgf21* (*n* = 12), AAV9‐CRISPRa‐sg*Fndc5* (*n* = 12), AAV9‐CRISPRa‐sg*Fgf21*+*Fndc5* (*n* = 12). In (C and D), chow (*n* = 10), *n* = 12 for other groups. In (E), chow (*n* = 10), AAV9‐CRISPRa‐sgSCR (*n* = 9), AAV9‐CRISPRa‐sg*Fgf21* (*n* = 8), AAV9‐CRISPRa‐sg*Fndc5* (*n* = 8), AAV9‐CRISPRa‐sg*Fgf21*+*Fndc5* (*n* = 9). In (F), chow (*n* = 8), AAV9‐CRISPRa‐sgSCR (*n* = 10), AAV9‐CRISPRa‐sg*Fgf21* (*n* = 9), AAV9‐CRISPRa‐sg*Fndc5* (*n* = 8), AAV9‐CRISPRa‐sg*Fgf21*+*Fndc5* (*n* = 10). In (G), chow (*n* = 5), AAV9‐CRISPRa‐sgSCR (*n* = 6), AAV9‐CRISPRa‐sg*Fgf21* (*n* = 5), AAV9‐CRISPRa‐sg*Fndc5* (*n* = 4), AAV9‐CRISPRa‐sg*Fgf21*+*Fndc5* (*n* = 5). In (H), chow (*n* = 3), AAV9‐CRISPRa‐sgSCR (*n* = 5), AAV9‐CRISPRa‐sg*Fgf21* (*n* = 4), AAV9‐CRISPRa‐sg*Fndc5* (*n* = 7), AAV9‐CRISPRa‐sg*Fgf21*+*Fndc5* (*n* = 6). *p < .05, **p < .01 and ***p < .001 versus the DIO mice received sgSCR virus. IPGTT, intraperitoneal glucose tolerance test. IPITT, intraperitoneal insulin tolerance test. DIO, diet‐induced obesity.

Obesity induces morphological changes, as well as pathological impairments, such as reduced insulin sensitivity. Insulin sensitivity can be indicated by reduced insulin tolerance and/or increased glucose tolerance, as evaluated by an IPITT and an IPGTT, respectively. To assess the effect of myokine activation on insulin sensitivity, DIO mice were randomly divided into four groups to receive the sgSCR, sg*Fgf21*, sg*Fndc5* or sg*Fgf21*+*Fndc5* virus. IPGTT and IPITT assays were performed to determine the baseline insulin sensitivity of mice in each group at the indicated time. The results showed that DIO mice in all groups had similar baseline insulin sensitivity, and both mice exhibited insulin insensitivity and were less glucose tolerant than chow mice (Figures 7A and B). Interestingly, 4 weeks after AAV injection, DIO mice with single or combined myokines activation showed greater glucose tolerance and insulin sensitivity comparable to chow mice (Figures 7C and D). These improvements were persistent and accumulative, as demonstrated in IPGTT and IPITT of chow mice and DIO mice even at 6‐ and 8‐weeks post AAV9 injection (Figures 7E–H). In conclusion, these results confirmed that in vivo CRISPRa‐based myokines activation by AAV9 can effectively ameliorate the glucose tolerance and insulin sensitivity of DIO mice over a relatively long period.

## 4 DISCUSSION

Obesity and T2D have become public health issues worldwide. In 2016, more than 1.9 billion adults, 18 years and older, were overweight. Of these, over 650 million were obese.^1^ Thrifty and efficient strategies for obesity treatments are in urgent need. Recently, numerous studies have identified skeletal muscle as a secretory organ. Skeletal muscles secrete myokines, which regulate various processes including adipocyte metabolism. In this study, we utilised a novel CRISPRa system to regulate the endogenous expression and secretion of two myokines of muscles in vitro and in vivo, ultimately ameliorating obesity through myokines–adipocytes interactions.

The endocrine function of myokines is implicated in body weight regulation, inflammation, insulin sensitivity, suppression of tumour growth and improvement of cognitive function.^44^ Myokines‐based therapies are considered to enable ‘exercise in a pill’ in the future and are conducted in laboratory investigations and clinical trials related to obesity, cancer, cardiovascular diseases and neurodegenerative diseases.^45^, ^46^, ^47^ In principle, this strategy is beneficial for multiorgan with few side effects. As mentioned above, FGF21 and Irisin are two myokines that play an important role in muscle‐adipocyte crosstalk and adipocytes browning. FGF21 was first identified in 2005 as a novel metabolic regulator with therapeutic effects in diabetes treatments.^48^ Induced by exercise, Irisin could promote the browning of white adipocytes, making it a potential target in improving obesity.^29^ In the past decade, much research has focused on these two targets to promote adipocytes browning and treat obesity effectively. Administration of the recombinant FGF21 protein improves insulin sensitivity and lowers body weight and blood glucose significantly in obese mice, rats and rhesus monkeys.^48^, ^49^, ^50^ However, the native FGF21 protein has poor pharmacokinetic properties, which narrowed its clinical applications. Multiple analogues or mimetics of FGF21 have been developed to solve this problem, some of which have completed phase I clinical trials with a noticeable improvement in obesity and T2D‐related symptoms in patients.^51^, ^52^ The purification and biological characterization of recombinant human Irisin was performed by Panati in 2018^53^; administration of recombinant Irisin also alleviates obesity and obesity‐related disorders in obese mice.^54^, ^55^ Besides the pharmacokinetic imperfections, recombinant myokines or analogues require periodic intake, which may be inconvenient and trigger immunoreaction due to the administration of exogenous proteins.^5^ Jimenez and colleagues^5^ performed AAV gene therapy to achieve sustaining liver‐specific FGF21 expression in vivo, which ultimately ameliorates obesity and insulin resistance. However, the two main safety concerns of gene overexpression remain challenging: excessive upregulation fold (usually more than hundreds, even thousands) and potential abnormal endogenous gene expression caused by random insertions of exogenous gene fragments into the genome.

CRISPRa‐based myokines activation could overcome barriers from periodic intake of recombinant myokines and uncontrolled overexpression of traditional gene therapy, providing a continuous, mutation‐independent, moderate, but effective activation approach. Moreover, the CRISPRa‐based activation at the transcriptional level produced an elevated expression of endogenous myokines, closer to natural states under physiological conditions. The CRISPRa system has been used to treat many diseases, including glioma, muscular dystrophy and triple‐negative breast cancer.^19^, ^56^, ^57^ To our knowledge, this study is the first to activate myokines to enhance myokines–adipocytes interactions using the CRISPRa system.

AAV is the most effective tool for in vivo gene delivery.^58^, ^59^ In this study, we use modified AAV9 to deliver the CRISPRa system. Using the reinvented AAV9 packaging system, we successfully generated the CRISPRa system to activate myokines in vivo. However, the CMV promotor we used can activate multiorgan myokines expression in a broad‐spectrum way, and the impacts of this comprehensive activation on vital organs, such as the brain and heart, remained unexplored. Yet hormones produced by different organs might not have similar physiological functions.^60^ Mice injected with AAVs in this study did not show a significant overall abnormality, though (Figures S6 and S9). A more accurate assessment should be performed further to assess the CMV promotor in this type of study. It is exciting that a family of AAV capsid variants with more muscle‐specific gene delivery across species has been developed recently.^61^ New AAVs systems with improved capacity and enhanced muscle specificity would benefit the future application of CRISPRa‐based gene therapy.

We also explored the possibility of jointly activating more than one gene to maximise efficiency. Our results showed that the CRISPRa system with sg*Fgf21+Fndc5* could upregulate the expression of *Fgf21* and *Fndc5* simultaneously. In vitro assays also indicate that coactivation of *Fgf21* and *Fndc5* in muscle cells is more efficient than single myokine activation in promoting adipocytes browning. Surprisingly, the same effect was not observed in vivo. Although *Fgf21* and *Fndc5* are all activated in DIO mice, those mice did not manifest a significantly better improvement than mice that received AAVs targeting a single myokine. This inconsistency might be due to the relatively lower titre of AAV9‐sgRNAs; in the coactivation group, the amount of each sgRNA was reduced by half. Further in vivo investigations, such as indirect calorimetry measurements, are needed to understand the precise mechanisms and the physiological significance of the induced adipocytes browning.

Besides the therapeutic effect, the biosafety of the CRISPRa system is an indispensable precondition for future clinical practices. In this study, we paid close attention to healthy C57BL/6 mice and HFD mice injected with AAVs, and we did not observe any differences or additional abnormalities in the mice. Although some concerns about AAV‐mediated gene therapy have been reported in recent years,^62^ AAV remains the safest tool for in vivo gene delivery and is widely used in a large number of clinical trials.^58^ The safety of the CRISPRa system consists of dCas9‐VP64 and sgRNA was also certified in muscles and adipocyte tissues in preclinical studies.^21^, ^22^, ^63^ Apart from ameliorating obesity, elevated serum FGF21 and Irisin levels are beneficial for hypothyroidism, polycystic ovary syndrome, Prader–Willi syndrome, and even can serve in the treatment of severe acute respiratory syndrome coronavirus 2 infection.^64^ On the basis of the above results, we believe that the in vivo CRISPRa system has adequate biosafety and further side‐by‐side comparison between different treatment approaches should be conducted in the future.

## 5 CONCLUSIONS

The CRISPRa system was utilised in this study to activate the expression and secretion of FGF21 and Irisin, which promotes adipocytes browning in vitro and in vivo, reduces body weight and body fat and improves HFD‐associated WAT hypertrophy, hepatic steatosis, inflammation and fibrosis of DIO mice in vivo. Our findings provide a novel strategy targeting myokines–adipocytes interactions for obesity treatment based on the CRISPRa system.

## FUNDING INFORMATION

This study was supported by the National Natural Science Foundation of China 81471283 (B. Z.) and the National Natural Science Foundation of China 82072795 (F. W.).

## CONFLICT OF INTEREST STATEMENT

The authors declare no competing interests

## CONSENT FOR PUBLICATION

Not applicable.

## ETHICS STATEMENT AND CONSENT TO PARTICIPATE

All experimental procedures were performed following the Guide for the Care and Use of Laboratory Animals and approved by the University Animal Welfare Committee, Tongji Medical College, Huazhong University of Science and Technology.

## Supporting information


**Figure S1: FGF21 and Irisin are expressed and secreted by muscle cells**. (A) Flow chart of myocyte differentiation. (B) Top‐bottom, representative bright field figures of different days that C2C12 myoblasts cultured with differentiation media. Half bottom, the fluorescent field of differentiated C2C12 myotubes (D7 post‐differentiation). (C, D) qRT‐PCR quantification of the mRNA expression of *Fgf21* and *Fndc5* in cell lysate during C2C12 differentiation. (E, F) The protein level of FGF21 and Irisin in conditioned media during C2C12 differentiation. Ponceau S staining was used as a loading control. (G, H) The protein levels of FGF21 and Irisin in cell lysates during C2C12 differentiation were examined by western blotting, and the blot quantification is on the right. FGF21 and Irisin were normalized to β‐actin (I, J).

**Figure S2: Activation of myokines in muscle cells can promote myocytes differentiation**. (A–D) qRT‐PCR quantification of skeletal muscle‐specific genes (*Myf5*, *Myg*, *Myd*, *Mck*) expression in C2C12 cells with *Fgf21*/*Fndc5* activation.

**Figure S3: Conditioned media of muscle cells with myokines activation inhibits fat accumulation of primary adipocytes *in vitro***. (A) Oli Red O staining of primary SVF‐derived adipocytes stimulated by conditioned media of C2C12 cells with activation of *Fgf21*(with or without FGFR1 inhibitor PD173074) and the relative intensity of lipid droplets (B) (n = 3). (C) Oli Red O staining of primary SVF‐derived adipocytes stimulated by conditioned media of C2C12 cells with activation of *Fndc5* (with or without Integrin αVβ5 inhibitor Cilengitide) and the relative intensity of lipid droplets (D) (n = 3). (E) Oli Red O staining of primary SVF‐derived adipocytes stimulated by conditioned media of C2C12 cells with activation of *Fgf21* and *Fndc5* (with or without FGFR1 inhibitor PD173074 and Integrin αVβ5 inhibitor Cilengitide) and the relative intensity of lipid droplets (F) (n = 3). Scale bars in A, C and E, 25 μm.

**Figure S4: Conditioned media of muscle cells with myokines activation promote adipocytes browning *in vitro***. (A) Western blot assay shows the level of UCP1 protein in 3T3‐L1 derived adipocytes stimulated by conditioned media of C2C12 cells with activation of *Fgf21*/*Fndc5*/*Fgf21*+*Fndc5* (with or without FGFR1 inhibitor PD173074 and Integrin αVβ5 inhibitor Cilengitide). Tubulin was used as an internal reference. (B) Western blot assay shows the level of UCP1 protein in primary adipocytes stimulated by conditioned media of C2C12 cells with activation of *Fgf21*/*Fndc5*/*Fgf21*+*Fndc5* (with or without FGFR1 inhibitor PD173074 and Integrin αVβ5 inhibitor Cilengitide). Tubulin was used as an internal reference. (C) Oxygen consumption assay shows the OCR of 3T3‐L1 derived adipocytes stimulated by conditioned media of C2C12 cells with activation of *Fgf21*/*Fndc5*/*Fgf21*+*Fndc5* (with or without FGFR1 inhibitor PD173074 and Integrin αVβ5 inhibitor Cilengitide).

**Figure S5: AAV package for AAV9‐CRISPRa system**. (A, B) AAV production methods. In the triple‐plasmid method, HEK293 cells expressing adenovirus E1a and E1b are co‐transfected with an adenovirus helper plasmid (pHelper), a rep/cap plasmid expressing AAV2 rep and AAV9 cap (pAAVrep2cap9), and the transgene plasmid carrying the rAAV‐transgene cassette (pAAV‐transgene). (C) The expression of AAV9‐CRISPRa‐EGFP and AAV9‐sgRNA‐tdTomato vector was detected in HEK293T. (D) DNA agarose gel electrophoresis shows successful packaging of AAV9‐CRISPRa. The left, middle and right primers used for PCR analysis are shown in Table S3.

**Figure S6: *In vivo* verification of AAV9 titer in healthy C57BL/6 mice**. (A) Schematic protocol for health C57BL/6 mice administered with 10^11^, 10^12^, 10^13^ vg/mouse of AAV9‐CRISPRa vectors. (n = 3). At 9 weeks post‐injection, mice were sacrificed for analysis. (B–F) The distribution of virus in the intestines, fore limb, hind limb, heart, lung, kidney, spleen, stomach, and liver were detected by fluorescence microscope.

**Figure S7: CRISPRa system effectively upregulates myokines expression and secretion of skeletal muscles in healthy C57BL/6 mice**. (A) Schematic protocol for this assay. (B, C) qRT‐PCR quantification of *Fgf21* and *Fndc5* activation in skeletal muscles of C57BL/6 mice injected with different viruses. (D, E) ELISA assay shows the upregulation of FGF21 and Irisin in the serum of C57BL/6 mice injected with different viruses.

**Figure S8: AAV9 can deliver the CRISPRa system into DIO mice**. (A–C) The virus distributions in the limb, intestines, heart, stomach, kidney, liver, lung, and spleen were detected by fluorescence microscope. GFP represents dCas9‐VP64 and RFP represents sgRNA.

**Figure S9: DIO‐mice induction**. (A) Pictures of 16‐week‐old chow and DIO littermates. (B) Weekly weight measurement during 10 weeks of HFD showed the ability of obese mice to gain weight (chow, n = 12; HFD, n = 48). (C) Cumulative food intake daily of chow and DIO mice. (D) Subscapular‐subcutaneous (back) and inguinal‐perigonadal (abdomen) fat deposits from 16‐week‐old chow and DIO mice. Asterisks point at fat depots. (E, F) NMR‐based body composition analysis revealed increased fat mass and lean mass in DIO mice at 16 weeks of age. (chow, n = 12, HFD, n = 48).

**Figure S10: DIO mice show low myokines expression and secretion along with low UCP1 expression**. (A, B) Relative mRNA levels of *Fgf21* and *Fndc5* in the muscle of chow‐fed and HFD mice measured by qRT‐PCR. (C, D) Serum FGF21 and Irisin levels measured by ELISA. (E) H&E staining of BAT sections (top) and immunostaining for the BAT marker UCP1 (bottom) of mice fed as chow and HFD. Scale bar, 100 μm. (F, G) Western blot analysis of UCP1 content in BAT of mice fed as chow and HFD (F). The blot quantification is on the right (G). UCP1 was normalized to Tubulin.

**Figure S11: DIO mice display WAT hypertrophy, hepatic steatosis, inflammation and larger adipocytes**. (A) H&E staining showed hepatic steatosis in 16‐week‐old DIO mice compared to control mice on a chow diet. Scale bar, 100 μm. (B‐E) qRT‐PCR quantification of the mRNA expression of *Col1a1* and the markers of inflammation *Tnfa*, *Adgre1*, and *Il1b* in the liver of 16‐week‐old chow‐ or HFD‐fed animals. (F, G) Representative images of the H&E staining of the eWAT and iWAT from chow‐fed or DIO mice at 16 weeks of age. Scale bar, 100 μm. (H, I) Morphometric analysis of the area of eWAT and iWAT adipocytes in chow‐fed or DIO mice. (n = 4, a total of 100 adipocytes were counted in each sample).

**Figure S12: *In vivo* CRISPRa‐based myokines activation improves HFD‐associated WAT hypertrophy in DIO mice**. (A, B) Morphometric analysis of the areas of eWAT and iWAT adipocytes in chow‐fed or DIO mice (n = 4, a total of 100 adipocytes were counted in each sample) corresponding to Figure 6C and D.

Supporting Information

Supporting Information

Supporting Information

Supporting Information

## Data Availability

All data generated or analysed during this study are included in this published article and its supplementary information files.
