# Supplementary figures and images for: CRISPRa‐based activation of Fgf21 and Fndc5 ameliorates obesity by promoting adipocytes browning
Source: Clin Transl Med. 2023 Jul 18;13(7):e1326. doi: 10.1002/ctm2.1326 (PMC10353577; doi:10.1002/ctm2.1326)

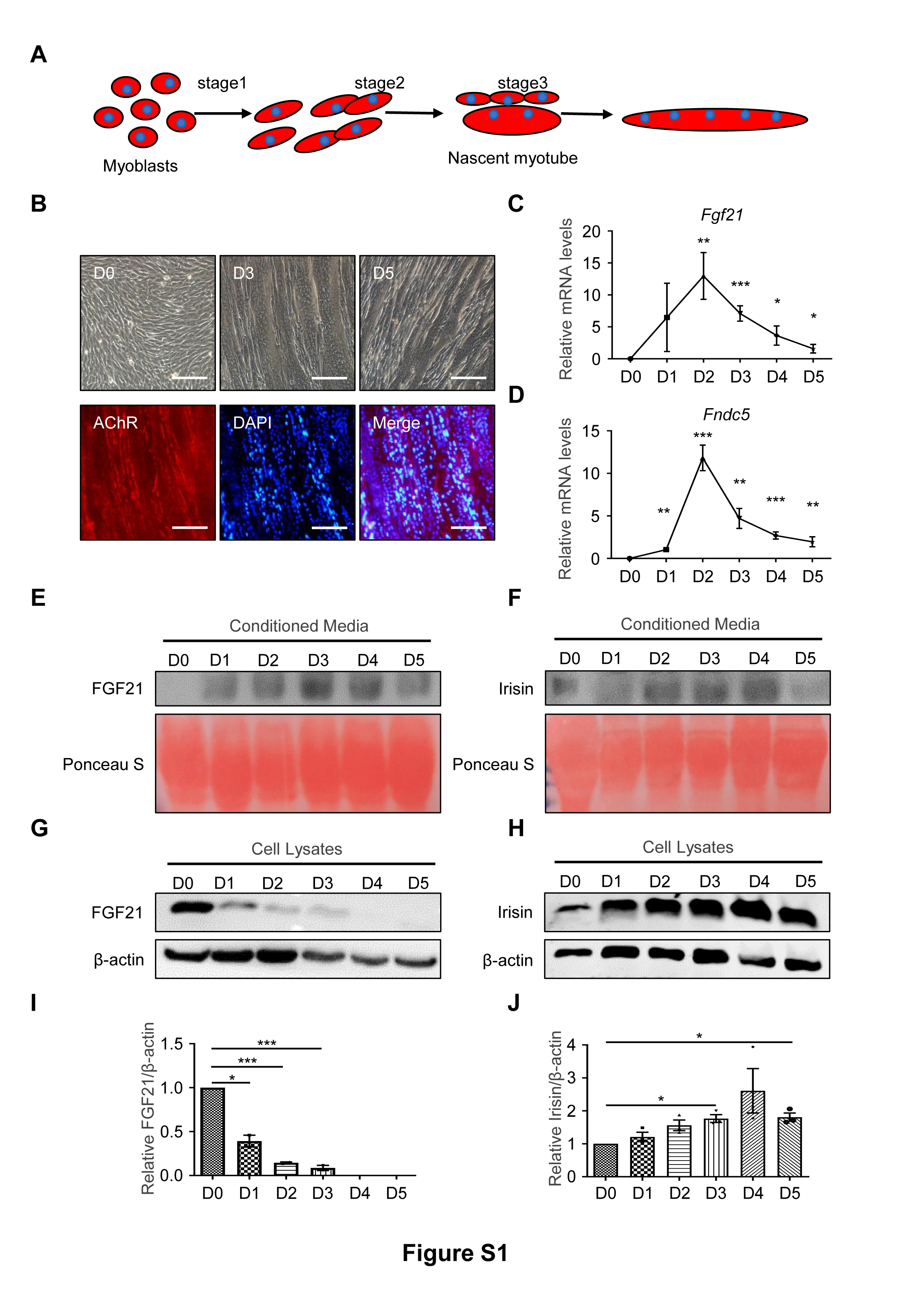

Supplement: Supplementary file 1 — Figure S1: FGF21 and Irisin are expressed and secreted by muscle cells. (A) Flow chart of myocyte differentiation. (B) Top‐bottom, representative bright field figures of different days that C2C12 myoblasts cultured with differentiation media. Half bottom, the fluorescent field of differentiated C2C12 myotubes (D7 post‐differentiation). (C, D) qRT‐PCR quantification of the mRNA expression of Fgf21 and Fndc5 in cell lysate during C2C12 differentiation. (E, F) The protein level of FGF21 and Irisin in conditioned media during C2C12 differentiation. Ponceau S staining was used as a loading control. (G, H) The protein levels of FGF21 and Irisin in cell lysates during C2C12 differentiation were examined by western blotting, and the blot quantification is on the right. FGF21 and Irisin were normalized to β‐actin (I, J). [file CTM2-13-e1326-s009.tif]

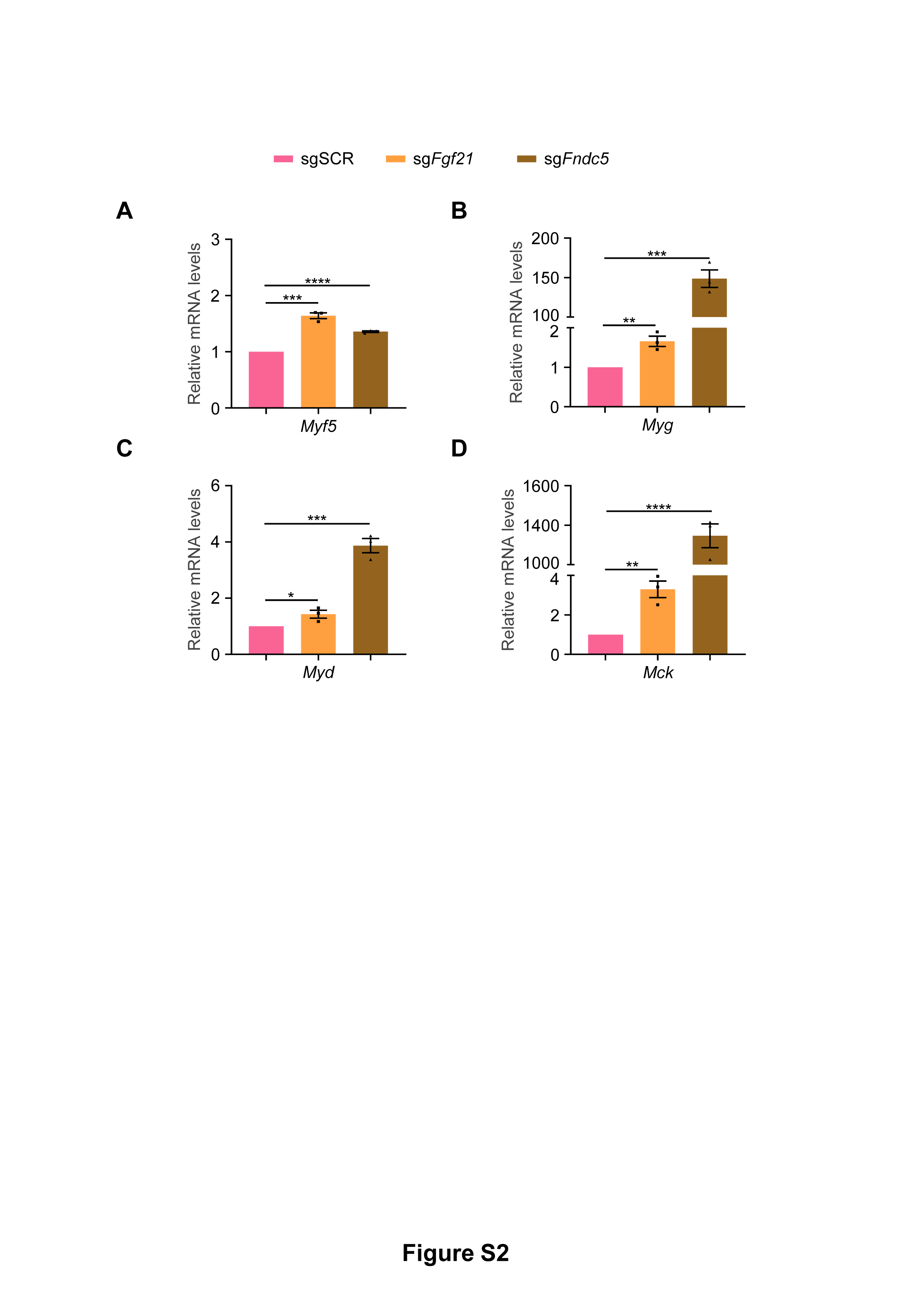

Supplement: Supplementary file 2 — Figure S2: Activation of myokines in muscle cells can promote myocytes differentiation. (A–D) qRT‐PCR quantification of skeletal muscle‐specific genes (Myf5, Myg, Myd, Mck) expression in C2C12 cells with Fgf21/Fndc5 activation. [file CTM2-13-e1326-s011.tif]

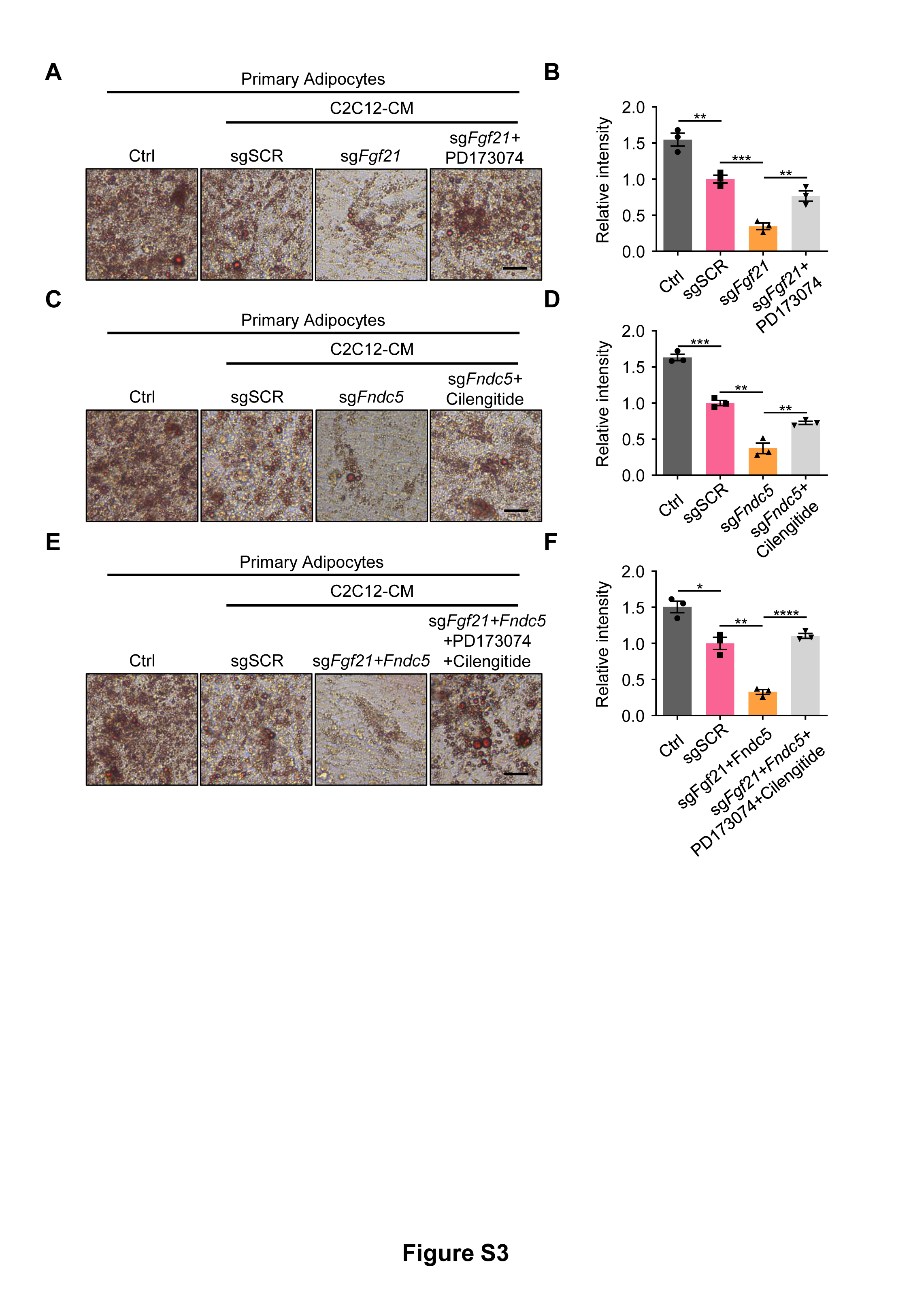

Supplement: Supplementary file 3 — Figure S3: Conditioned media of muscle cells with myokines activation inhibits fat accumulation of primary adipocytes in vitro . (A) Oli Red O staining of primary SVF‐derived adipocytes stimulated by conditioned media of C2C12 cells with activation of Fgf21(with or without FGFR1 inhibitor PD173074) and the relative intensity of lipid droplets (B) (n = 3). (C) Oli Red O staining of primary SVF‐derived adipocytes stimulated by conditioned media of C2C12 cells with activation of Fndc5 (with or without Integrin αVβ5 inhibitor Cilengitide) and the relative intensity of lipid droplets (D) (n = 3). (E) Oli Red O staining of primary SVF‐derived adipocytes stimulated by conditioned media of C2C12 cells with activation of Fgf21 and Fndc5 (with or without FGFR1 inhibitor PD173074 and Integrin αVβ5 inhibitor Cilengitide) and the relative intensity of lipid droplets (F) (n = 3). Scale bars in A, C and E, 25 μm. [file CTM2-13-e1326-s012.tif]

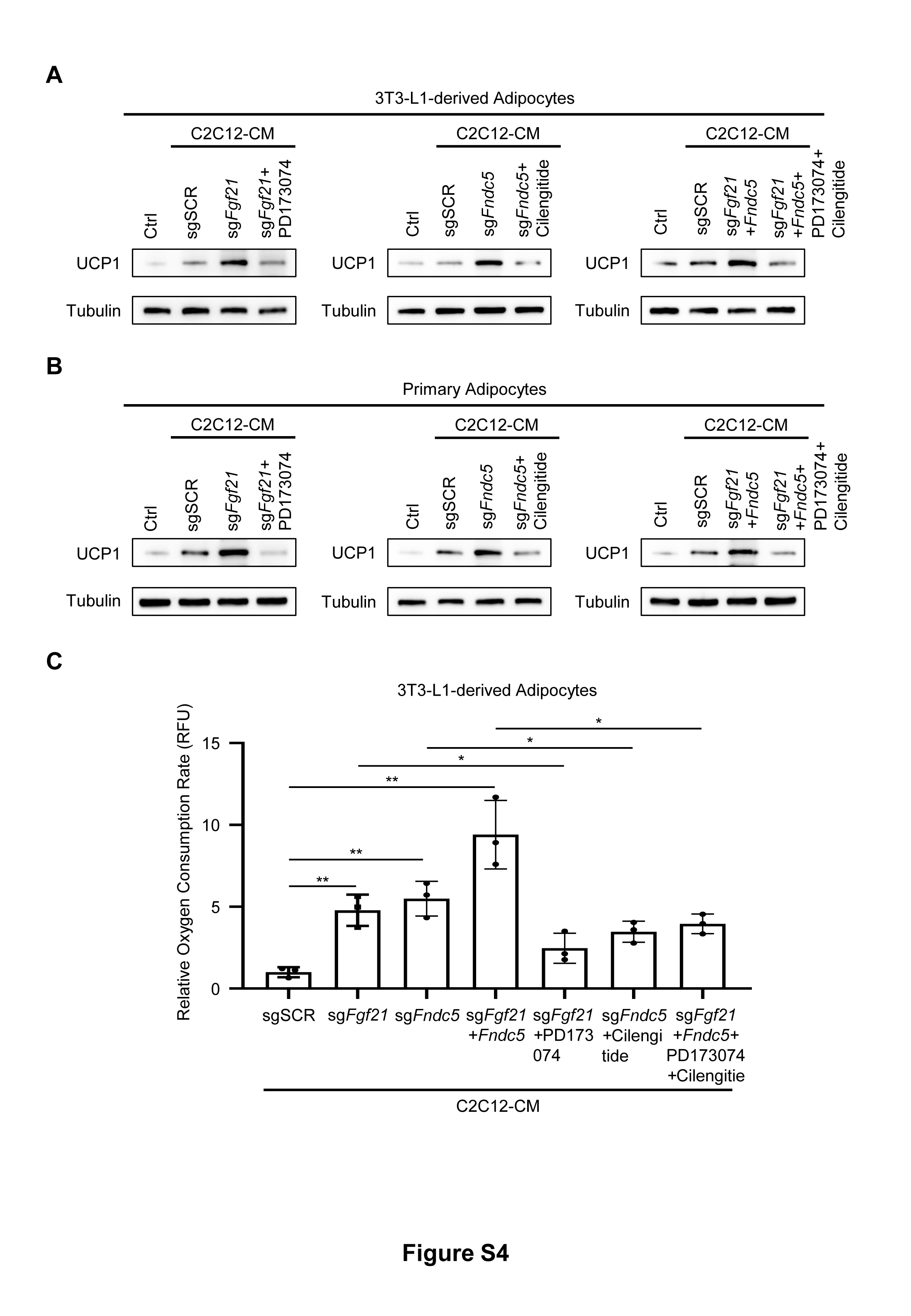

Supplement: Supplementary file 4 — Figure S4: Conditioned media of muscle cells with myokines activation promote adipocytes browning in vitro . (A) Western blot assay shows the level of UCP1 protein in 3T3‐L1 derived adipocytes stimulated by conditioned media of C2C12 cells with activation of Fgf21/Fndc5/Fgf21+Fndc5 (with or without FGFR1 inhibitor PD173074 and Integrin αVβ5 inhibitor Cilengitide). Tubulin was used as an internal reference. (B) Western blot assay shows the level of UCP1 protein in primary adipocytes stimulated by conditioned media of C2C12 cells with activation of Fgf21/Fndc5/Fgf21+Fndc5 (with or without FGFR1 inhibitor PD173074 and Integrin αVβ5 inhibitor Cilengitide). Tubulin was used as an internal reference. (C) Oxygen consumption assay shows the OCR of 3T3‐L1 derived adipocytes stimulated by conditioned media of C2C12 cells with activation of Fgf21/Fndc5/Fgf21+Fndc5 (with or without FGFR1 inhibitor PD173074 and Integrin αVβ5 inhibitor Cilengitide). [file CTM2-13-e1326-s015.tif]

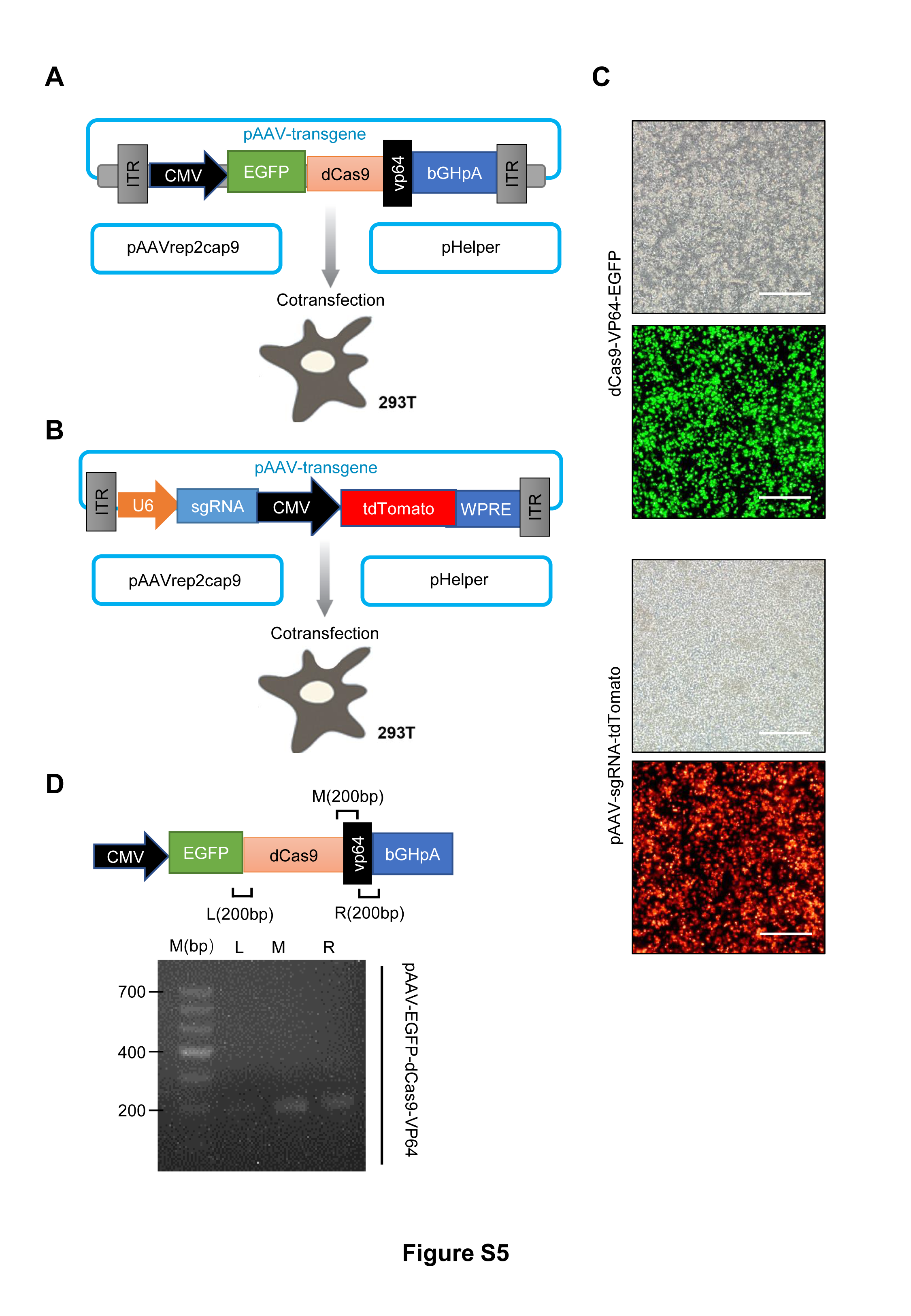

Supplement: Supplementary file 5 — Figure S5: AAV package for AAV9‐CRISPRa system. (A, B) AAV production methods. In the triple‐plasmid method, HEK293 cells expressing adenovirus E1a and E1b are co‐transfected with an adenovirus helper plasmid (pHelper), a rep/cap plasmid expressing AAV2 rep and AAV9 cap (pAAVrep2cap9), and the transgene plasmid carrying the rAAV‐transgene cassette (pAAV‐transgene). (C) The expression of AAV9‐CRISPRa‐EGFP and AAV9‐sgRNA‐tdTomato vector was detected in HEK293T. (D) DNA agarose gel electrophoresis shows successful packaging of AAV9‐CRISPRa. The left, middle and right primers used for PCR analysis are shown in Table S3. [file CTM2-13-e1326-s016.tif]

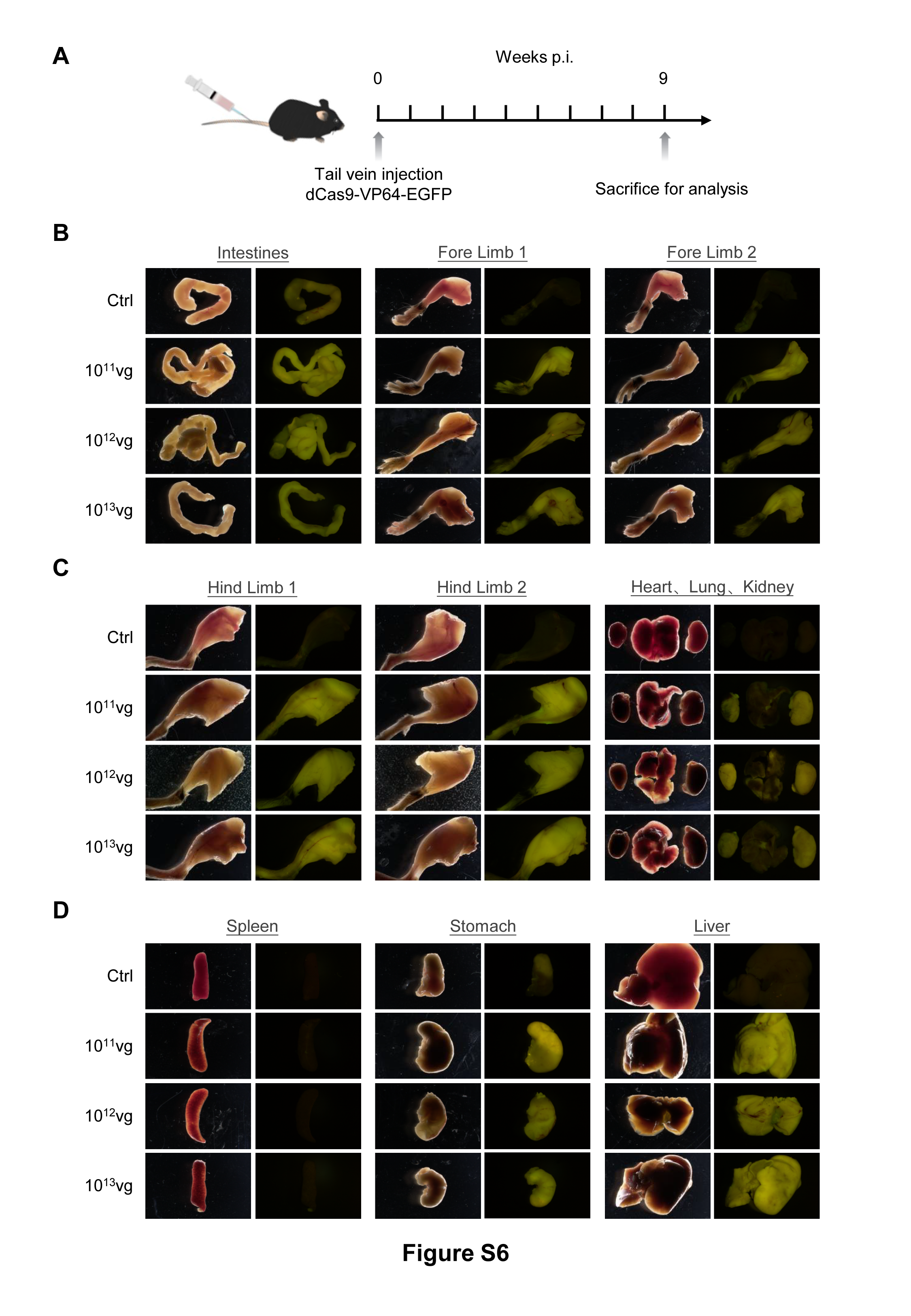

Supplement: Supplementary file 6 — Figure S6: In vivo verification of AAV9 titer in healthy C57BL/6 mice. (A) Schematic protocol for health C57BL/6 mice administered with 1011, 1012, 1013 vg/mouse of AAV9‐CRISPRa vectors. (n = 3). At 9 weeks post‐injection, mice were sacrificed for analysis. (B–F) The distribution of virus in the intestines, fore limb, hind limb, heart, lung, kidney, spleen, stomach, and liver were detected by fluorescence microscope. [file CTM2-13-e1326-s006.tif]

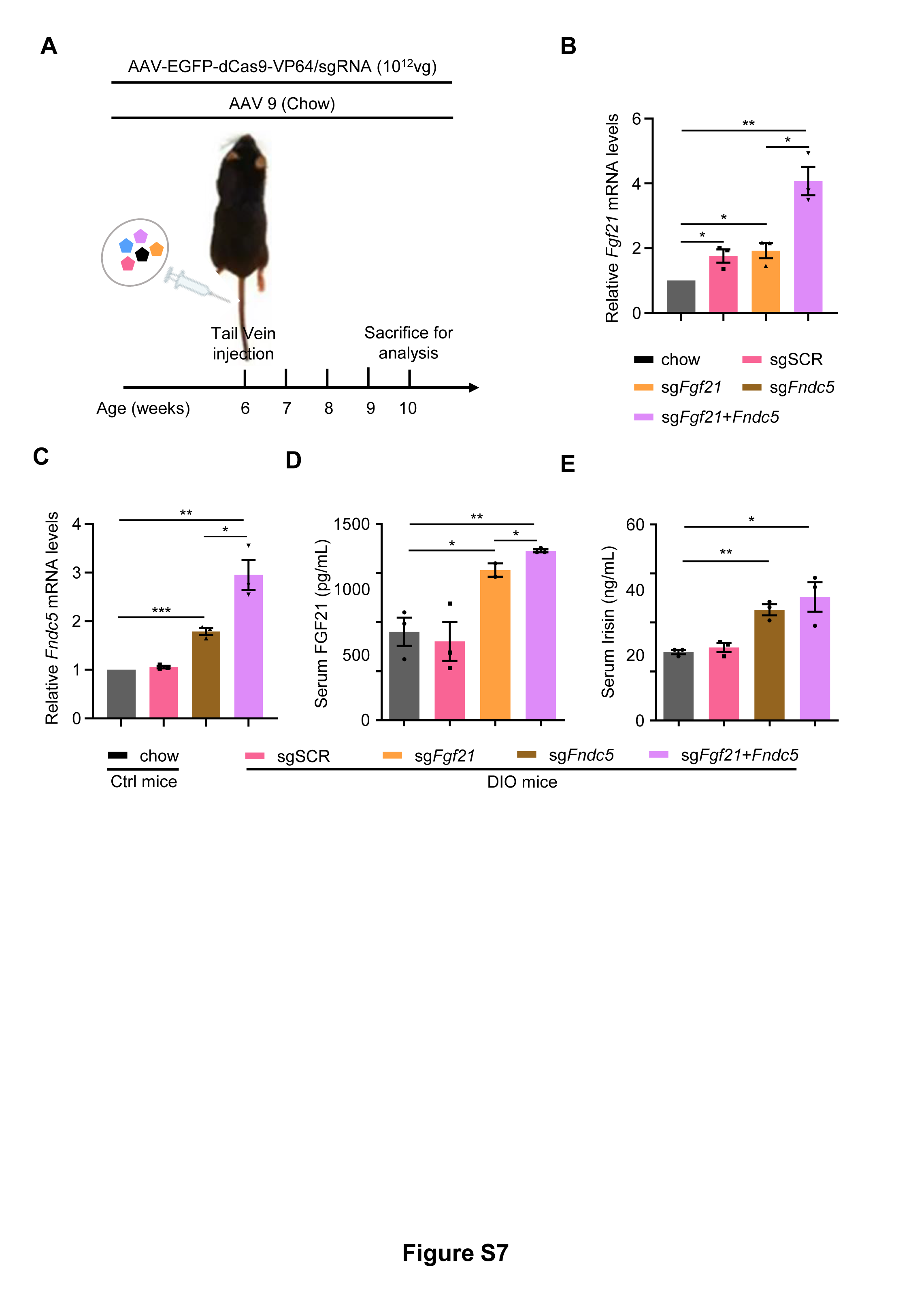

Supplement: Supplementary file 7 — Figure S7: CRISPRa system effectively upregulates myokines expression and secretion of skeletal muscles in healthy C57BL/6 mice. (A) Schematic protocol for this assay. (B, C) qRT‐PCR quantification of Fgf21 and Fndc5 activation in skeletal muscles of C57BL/6 mice injected with different viruses. (D, E) ELISA assay shows the upregulation of FGF21 and Irisin in the serum of C57BL/6 mice injected with different viruses. [file CTM2-13-e1326-s002.tif]

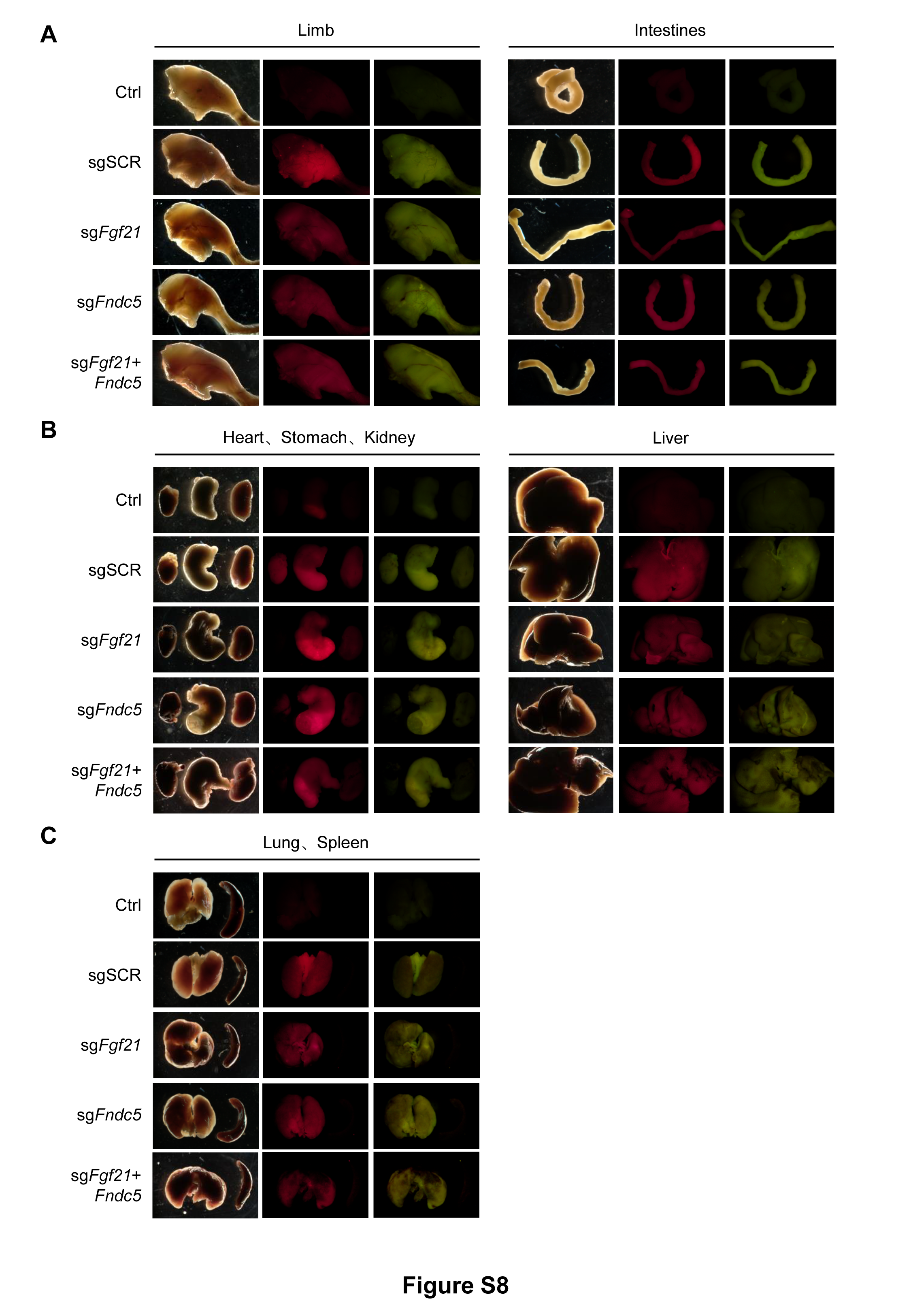

Supplement: Supplementary file 8 — Figure S8: AAV9 can deliver the CRISPRa system into DIO mice. (A–C) The virus distributions in the limb, intestines, heart, stomach, kidney, liver, lung, and spleen were detected by fluorescence microscope. GFP represents dCas9‐VP64 and RFP represents sgRNA. [file CTM2-13-e1326-s004.tif]

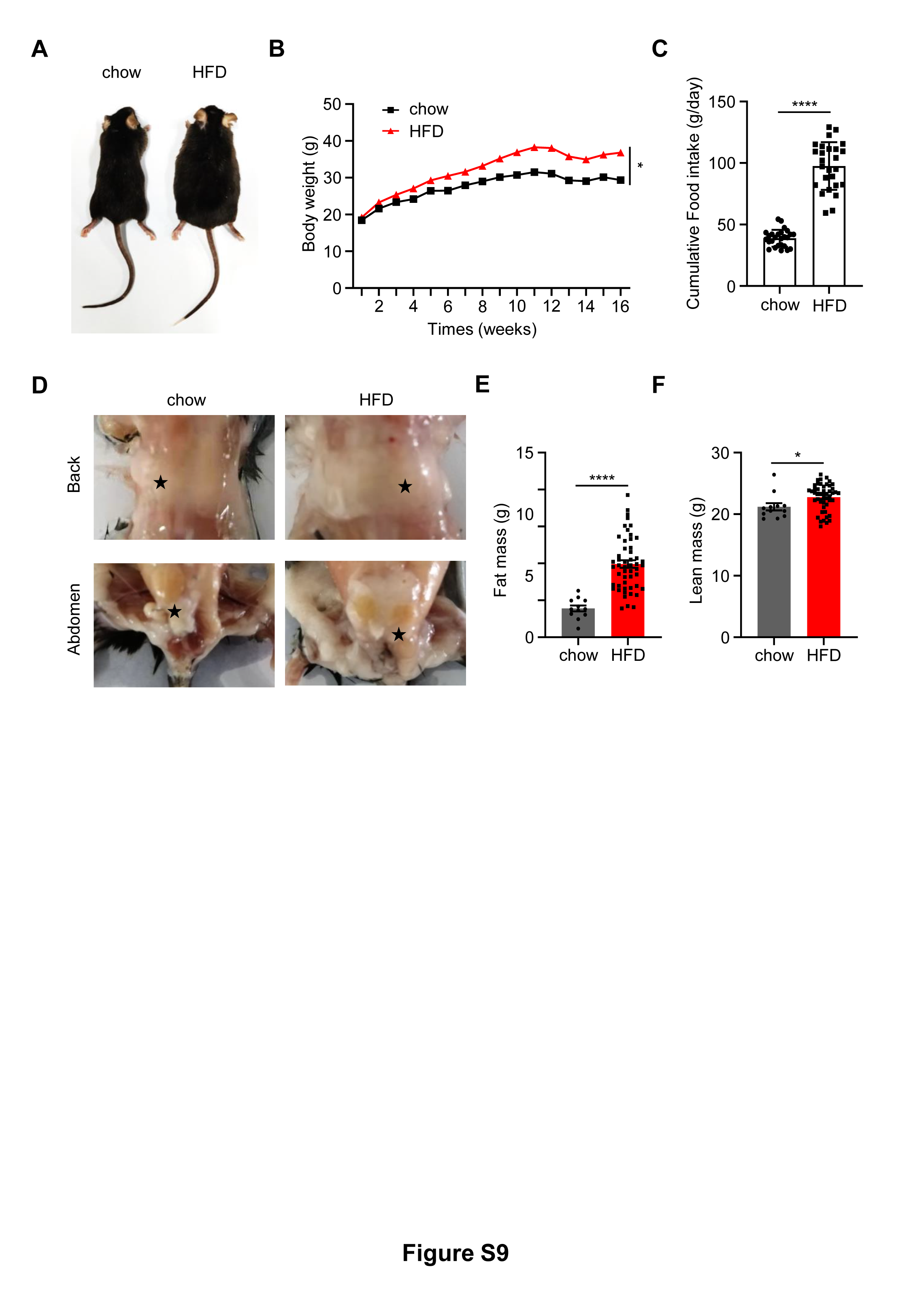

Supplement: Supplementary file 9 — Figure S9: DIO‐mice induction. (A) Pictures of 16‐week‐old chow and DIO littermates. (B) Weekly weight measurement during 10 weeks of HFD showed the ability of obese mice to gain weight (chow, n = 12; HFD, n = 48). (C) Cumulative food intake daily of chow and DIO mice. (D) Subscapular‐subcutaneous (back) and inguinal‐perigonadal (abdomen) fat deposits from 16‐week‐old chow and DIO mice. Asterisks point at fat depots. (E, F) NMR‐based body composition analysis revealed increased fat mass and lean mass in DIO mice at 16 weeks of age. (chow, n = 12, HFD, n = 48). [file CTM2-13-e1326-s010.tif]

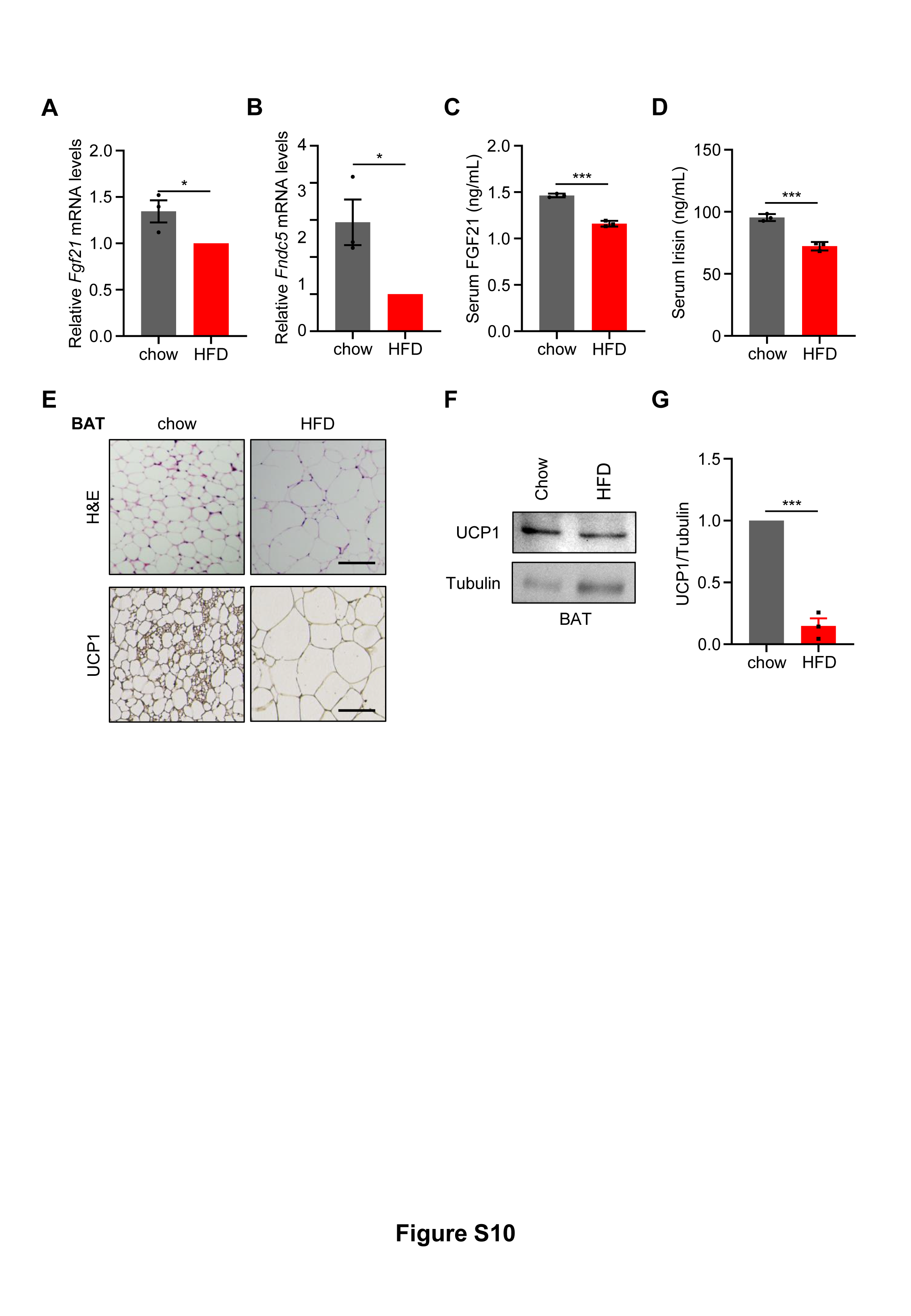

Supplement: Supplementary file 10 — Figure S10: DIO mice show low myokines expression and secretion along with low UCP1 expression. (A, B) Relative mRNA levels of Fgf21 and Fndc5 in the muscle of chow‐fed and HFD mice measured by qRT‐PCR. (C, D) Serum FGF21 and Irisin levels measured by ELISA. (E) H&E staining of BAT sections (top) and immunostaining for the BAT marker UCP1 (bottom) of mice fed as chow and HFD. Scale bar, 100 μm. (F, G) Western blot analysis of UCP1 content in BAT of mice fed as chow and HFD (F). The blot quantification is on the right (G). UCP1 was normalized to Tubulin. [file CTM2-13-e1326-s008.tif]

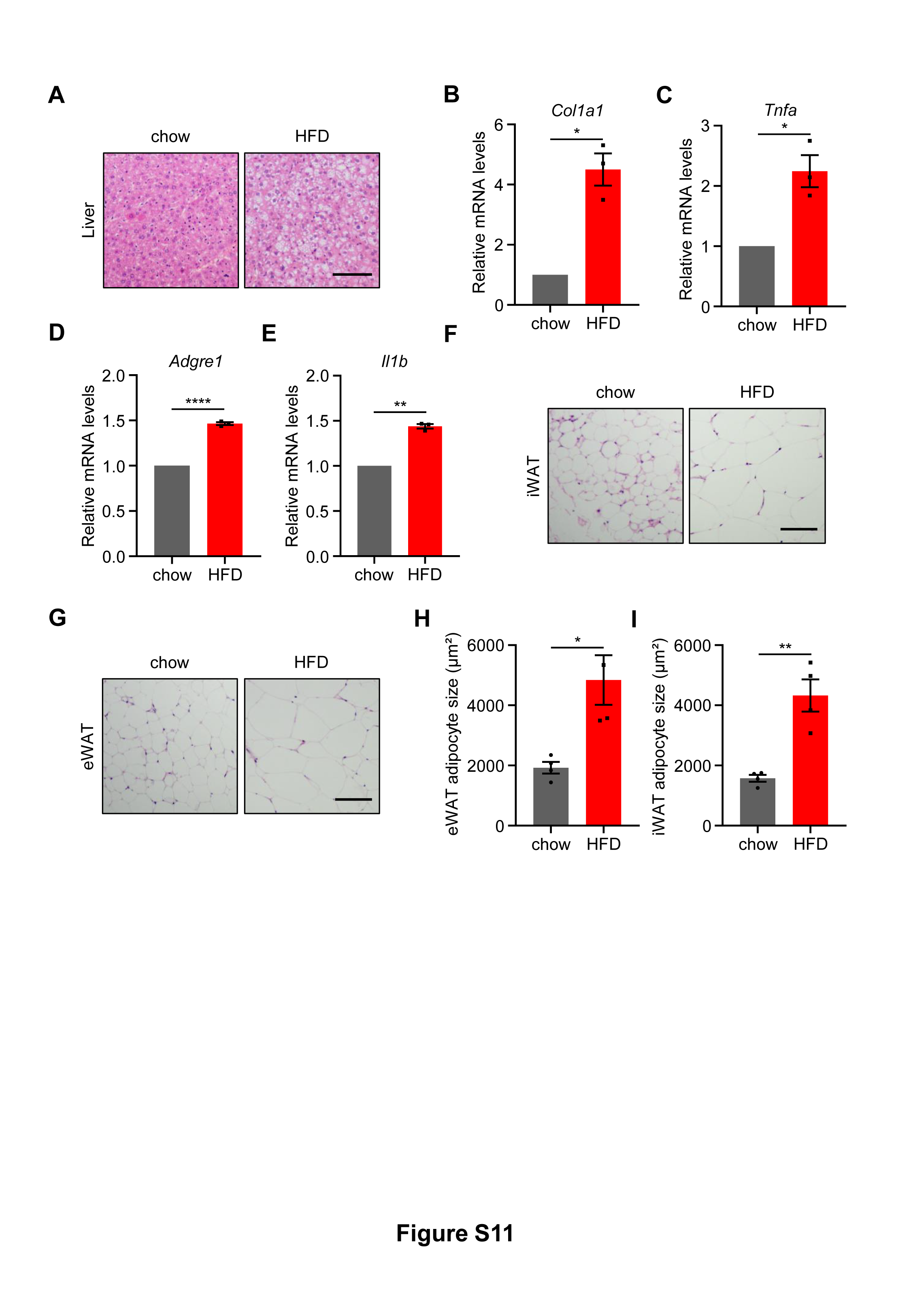

Supplement: Supplementary file 11 — Figure S11: DIO mice display WAT hypertrophy, hepatic steatosis, inflammation and larger adipocytes. (A) H&E staining showed hepatic steatosis in 16‐week‐old DIO mice compared to control mice on a chow diet. Scale bar, 100 μm. (B‐E) qRT‐PCR quantification of the mRNA expression of Col1a1 and the markers of inflammation Tnfa, Adgre1, and Il1b in the liver of 16‐week‐old chow‐ or HFD‐fed animals. (F, G) Representative images of the H&E staining of the eWAT and iWAT from chow‐fed or DIO mice at 16 weeks of age. Scale bar, 100 μm. (H, I) Morphometric analysis of the area of eWAT and iWAT adipocytes in chow‐fed or DIO mice. (n = 4, a total of 100 adipocytes were counted in each sample). [file CTM2-13-e1326-s007.tif]

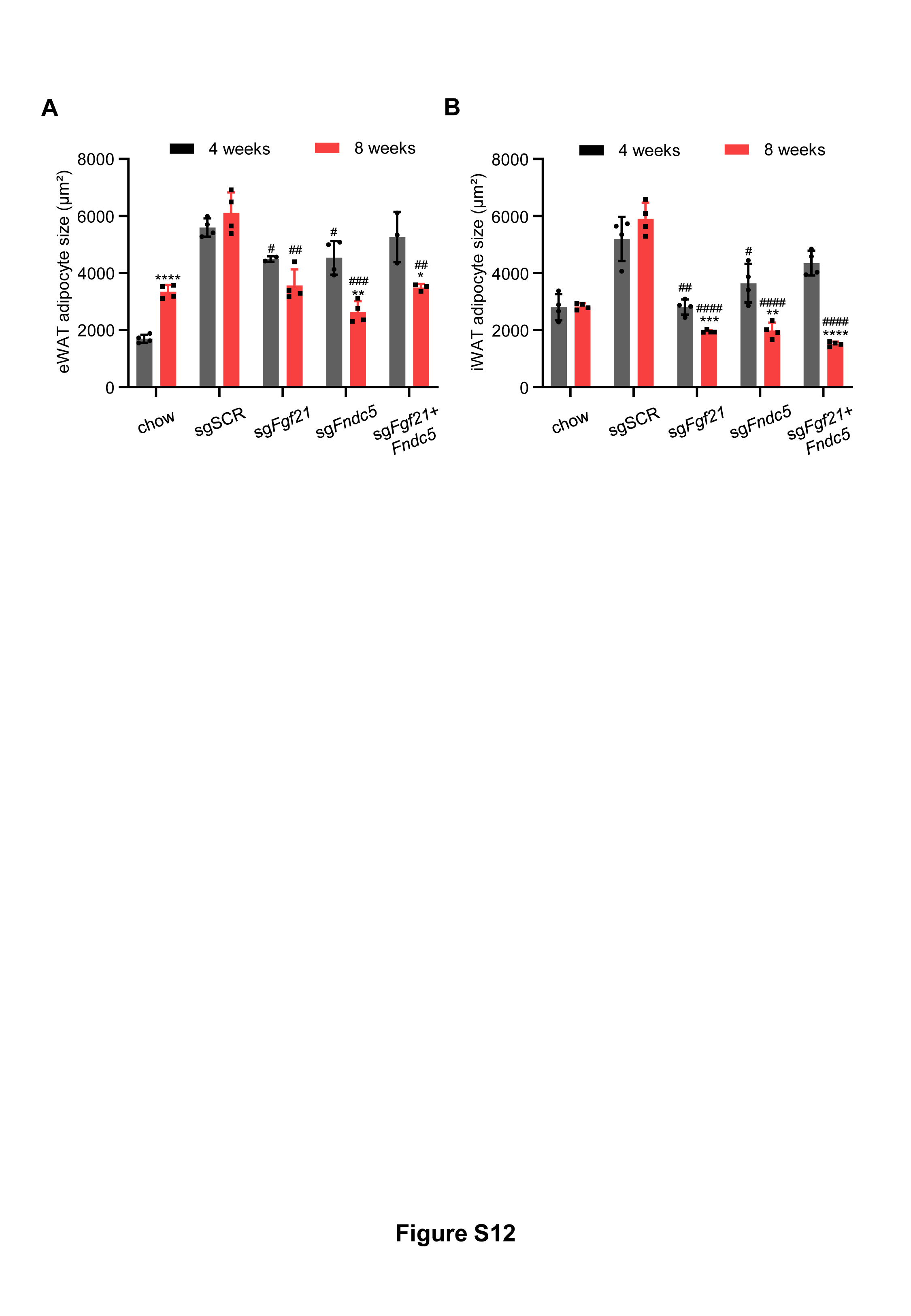

Supplement: Supplementary file 12 — Figure S12: In vivo CRISPRa‐based myokines activation improves HFD‐associated WAT hypertrophy in DIO mice. (A, B) Morphometric analysis of the areas of eWAT and iWAT adipocytes in chow‐fed or DIO mice (n = 4, a total of 100 adipocytes were counted in each sample) corresponding to Figure 6C and D. [file CTM2-13-e1326-s014.tif]

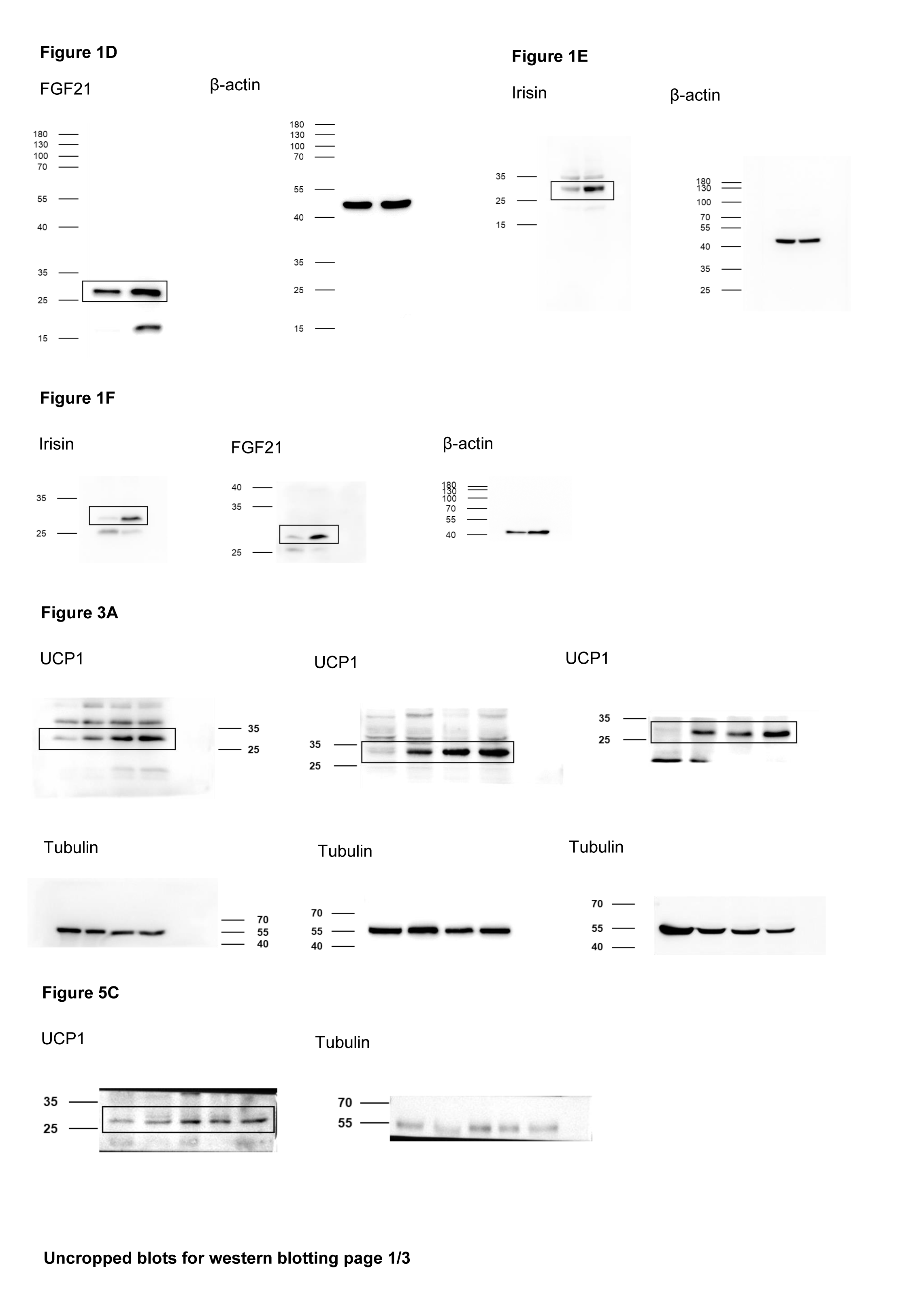

Supplement: Supplementary file 13 — Supporting Information [file CTM2-13-e1326-s005.tif]

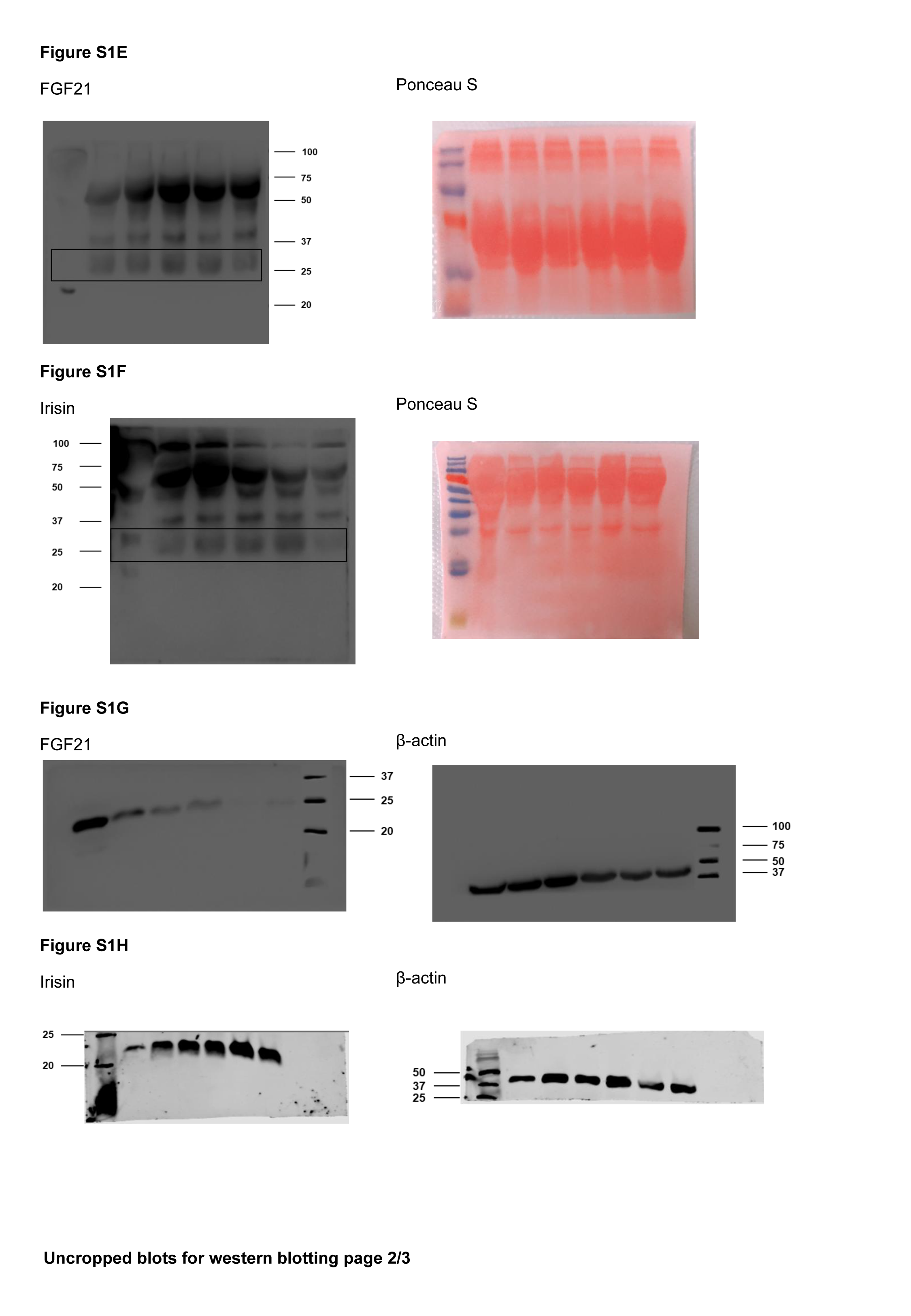

Supplement: Supplementary file 14 — Supporting Information [file CTM2-13-e1326-s013.tif]

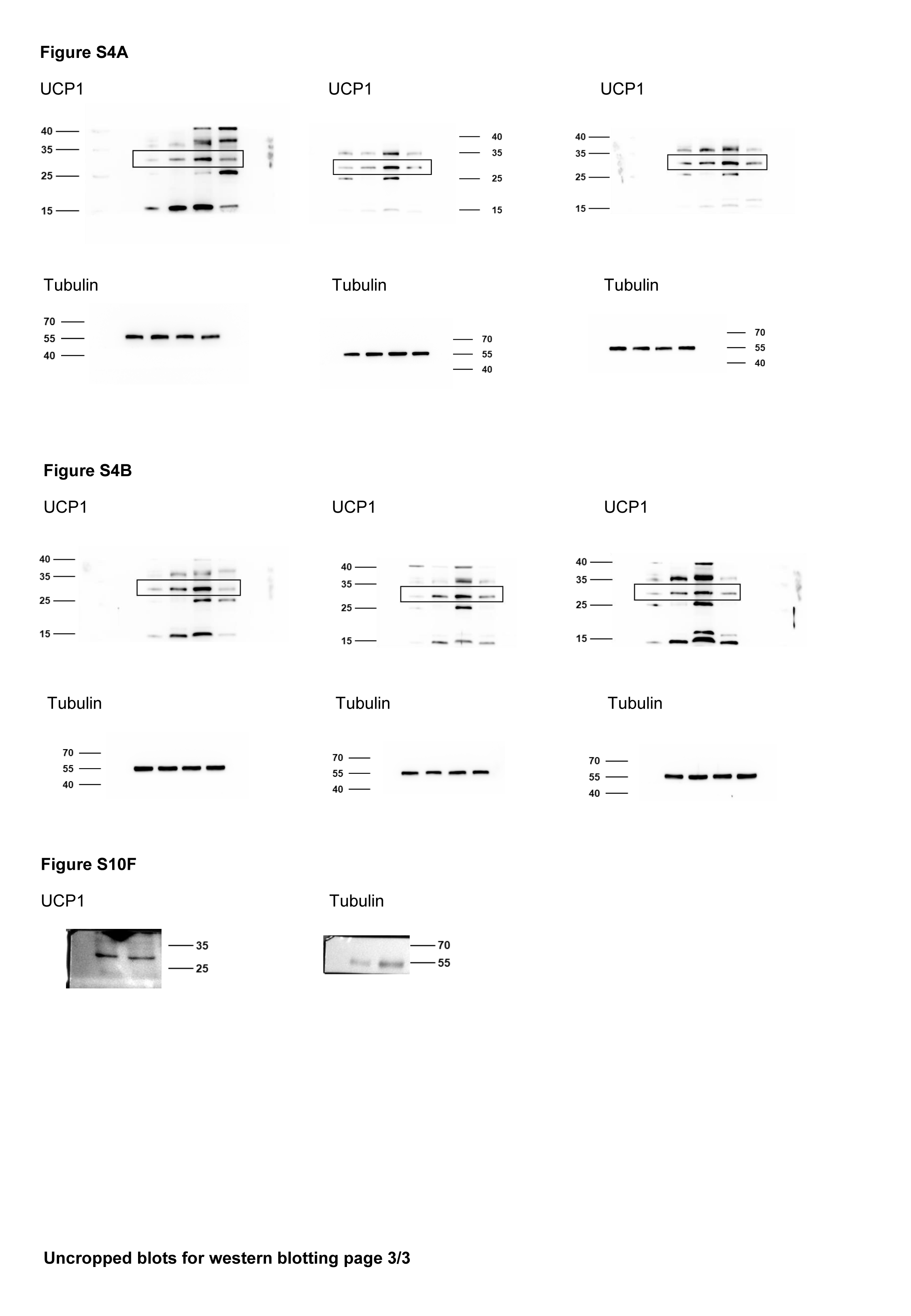

Supplement: Supplementary file 15 — Supporting Information [file CTM2-13-e1326-s003.tif]
