# Supplementary material for: CRISPRa‐based activation of Fgf21 and Fndc5 ameliorates obesity by promoting adipocytes browning
Source: Clin Transl Med. 2023 Jul 18;13(7):e1326. doi: 10.1002/ctm2.1326 (PMC10353577; doi:10.1002/ctm2.1326)
Supplement: Supplementary file 16 — Supporting Information [file CTM2-13-e1326-s001.docx]

**Supplementary Tables**

**Table S1 Plasmids**

| Plasmid | SOURCE | IDENTIFIER |
| --- | --- | --- |
| psPAX2 | Addgene | #12260 |
| pMD2.G | Addgene | #12259 |
| pU6-sgRNA EF1Alpha-puro-T2A-BFP | Addgene | #60955 |
| dCas9-VP64-EGFP | Addgene | #61422 |
| AAV-sgRNA backbone-hSyn-tdTomato  AAV-cDNA6-EGFP-Tav2A-V5His  pHelper  AAV9 | Kind gift from Dr. Jiang^1^  Our Lab  Kind gift from Dr. Jiang  Kind gift from Dr. Jiang | N/A  N/A  N/A  N/A |

1. Modified from the original plasmid (Elife. 2022. 11: e70664)

**Table S2 Primers used for vector construction**

| **Plasmid** | **Insert** | **Forward primer** | **Reverse primer** |
| --- | --- | --- | --- |
| pU6-sg*Fgf21*-EF1Alpha-puro-T2A-BFP | sg*Fgf21*#1 | GGAGAACCACCTTGTTGGATCAG  GGCTGCGCTCCGTTCGTTTAA  GAGCTATGCTGGAAACAGCA | CTAGTACTCGAGAAAAAAAGCACCGACTCGGTGCCAC |
|  | sg*Fgf21*#2 | GGAGAACCACCTTGTTGGCAT  CAGGGCTGCGCTCCGTTGTTT  AAGAGCTATGCTGGAAACAGCA |  |
|  | sg*Fgf21*#3 | GGAGAACCACCTTGTTGGTTGG  GGGTCAAGTCCGGCAGGTTTAA  GAGCTATGCTGGAAACAGCA |  |
| pU6-sg*Fndc5*-EF1Alpha-puro-T2A-BFP | sg*Fndc5*#1 | GGAGAACCACCTTGTTGGGAAC  CAGCAGCTGCGAACGGTTTAAG  AGCTATGCTGGAAACAGCA |  |
|  | sg*Fndc5*#2 | GGAGAACCACCTTGTTGGCGTG  AGTGCGAGCGGTCACGTTTAAG  AGCTATGCTGGAAACAGCA |  |
|  | sg*Fndc5*#3 | GGAGAACCACCTTGTTGGAACC  AGCAGCTGCGAACGGGTTTAAG  AGCTATGCTGGAAACAGCA |  |
| pU6-sgSCR-EF1Alpha-puro-T2A-BFP | sgSCR | GGAGAACCACCTTGTTGGTGTA  GTTCGACCATTCGTGGTTTAAG  AGCTATGCTGGAAACAGCA |  |
| dCas9-VP64-Neo-T2A-EGFP | Neo | ACCTGGACATGCTGATTAACGC  TAGCATGATTGAACAAGATGGA  TTGCAC | CCTCTGCCCTCTCCACTGCCGCTAGCGAAGAACTCGTCAAGAAGGCGA |
| AAV-sgRNA-CMV-tdTomato | CMV | TCTAGAGGCCTTGACATTGATTATTGAC | GGTACCAGCTCTGCTTATATAGACCTCC |
| AAV-sg*Fgf21*-CMV-  tdTomato | sg*Fgf21* | CACCGCATCAGGGCTGCGCTCCGTT | AAACAACGGAGCGCAGCCCTGATGC |
| AAV-sg*Fndc5*-CMV-  tdTomato | sg*Fndc5* | CACCGCCTAGCCACAGGCGGAGCG | AAACCGCTCCGCCTGTGGCTAGGC |
| AAV-sgSCR-CMV-  tdTomato | sgSCR | CACCGTGTAGTTCGACCATTCGTG | AAACCACGAATGGTCGAACTACAC |
| AAV-cDNA6-EGFP-dCas9-VP64-Tav2A-V5His | dCas9-VP64 | GACGAGCTGTACAAGGGATCCCCCAAGAAGAAGAGAAAGGT | GCCCTCTAGACTCGAGCGGCCGCCAGCATGTCCAGGTCGAAAT |
| pU6-mU6-sg*Fndc5*-U6-sg*Fgf21*-EF1Alpha-puro-T2A-BFP | U6-sg*Fgf21* | ACCGAGTCGGTGVTTTTTTTTCTCGAGAACTCCATCACTAGGGGTTCCT | GCGGTAATACGGTTATCCACGCGGCCGCCTAGTCAATAATCAATGTCAAGGCC |
| AAV-U6-sg*Fgf21*-mU6-sg*Fndc5*-CMV-tdTomato | mU6-sg*Fndc5* | CACCGAGTCGGTGCTTTTTTAACAGCACAAAAGGAAACTCACCCT | CAATAATCAATGTCAAGGCCCGGCCGCCTAATGGATCCTAGTACT |

**Table S3** **Primers used for PCR analysis**

| **Plasmid** | **sites** | **Forward primer** | **Reverse primer** |
| --- | --- | --- | --- |
| AAV-cDNA6-EGFP-dCas9-VP64-Tav2A-V5His | Left | AGCCCCAAGAAGAAGAGAAAGGT | TTCGCCGCTGTCGAACAGCA |
|  | Middle | ACCTAAGAAAAAGAGGAAGGTGG | TTAATCAGCATGTCCAGGTC |
|  | Right | CAGGGCGATAGCCTGCACGA | ATCCGCTTCATTCTCTCGCGG |

**Table S4 Primers used for real-time PCR assays**

| **Gene** | **Forward primer** | **Reverse primer** |
| --- | --- | --- |
| *Gapdh* | TGACCACAGTCCATGCCATC | GACGGACACATTGGGGGTAG |
| *Fgf21* | CTGCTGGGGGTCTACCAAG | CTGCGCCTACCACTGTTCC |
| *Fndc5* | CACGCGAGGCTGAAAAGATG | CCTTGTTGTTATTGGGCTCGTT |
| *Myd* | CGCCACTCCGGGACATAG | GAAGTCGTCTGCTGTCTCAAAGG |
| *Myg* | AGCGCAGGCTCAAGAAAGTGAATG | CTGTAGGCGCTCAATGTACTGGAT |
| *Mck* | GCAAGCACCCCAAGTTTGA | ACCTGTGCCGCGCTTCT |
| *Myf5* | CAGCCCCACCTCCAACTG | GGGACCAGACAGGGCTGTTA |
| *Ucp1* | AGGCTTCCAGTACCATTAGGT | CTGAGTGAGGCAAAGCTGATTT |
| *Ppargc1a* | CCCTGCCATTGTTAAGACC | TGCTGCTGTTCCTGTTTTC |
| *Cidea* | TGCTCTTCTGTATCGCCCAGT | GCCGTGTTAAGGAATCTGCTG |
| *Elovl3* | TTCTCACGCGGGTTAAAAATGG | GAGCAACAGCTAGACGACCAC |
| *Cycs* | GCAAGCATAAGACTGGACCAAA | TTGTTGGCATCTGTGTAAGAGAATC |
| *Cox8b* | GAACCATGAAGCCAACGACT | GCGAAGTTCACAGTGGTCC |
| *Cox7a1* | CAGCGTCATGGTCAGTCTGT | AGAAAACCGTGTGGCAGAGA |
| *Tfap2a* | GGAACACGTCGTGGGATAATG | GGAACACGTCGTGGGATAATG |
| *Adipoq* | GCACTGGCAAGTTCTACTGCAA | GTAGGTGAAGAGAACGGCCTTGT |
| *Col1a1* | GACTGGAAGAGCGGAGAGTA | CCTTGATGGCGTCCAGGTT |
| *Il1b* | TGTAATGAAAGACGGCACACC | TCTTCTTTGGGTATTGCTTGG |
| *Adgre1* | CTTTGGCTATGGGCTTCCAGTC | GCAAGGAGGACAGAGTTTATC |
| *Tnfa* | CATCTTCTCAAAATTCGAGTGACAA | TGGGAGTAGACAAGGTACAACCC |
| *ITR* | GGAACCCCTAGTGATGGAGTT | CGGCCTCAGTGAGCGA |
